# Blue-Print Autophagy in 2020: A Critical Review

**DOI:** 10.3390/md18090482

**Published:** 2020-09-21

**Authors:** Sergey A. Dyshlovoy

**Affiliations:** Laboratory of Pharmacology, A.V. Zhirmunsky National Scientific Center of Marine Biology, Far Eastern Branch, Russian Academy of Sciences, 690041 Vladivostok, Russian; dyshlovoy@gmail.com

**Keywords:** autophagy, macroautophagy, marine natural compounds, cancer, neurodegenerative disorders

## Abstract

Autophagy is an elegant and complex biological process that has recently attracted much attention from the scientific community. The compounds which are capable of control and modulation of this process have a promising potential as therapeutics for a number of pathological conditions, including cancer and neurodegenerative disorders. At the same time, due to the relatively young age of the field, there are still some pitfalls in the autophagy monitoring assays and interpretation of the experimental data. This critical review provides an overview of the marine natural compounds, which have been reported to affect autophagy. The time period from the beginning of 2016 to the middle of 2020 is covered. Additionally, the published data and conclusions based on the experimental results are re-analyzed with regard to the guidelines developed by Klionsky and colleagues (Autophagy. 2016; 12(1): 1–222), which are widely accepted by the autophagy research community. Remarkably and surprisingly, more than half of the compounds reported to be autophagy activators or inhibitors could not ultimately be assigned to either category. The experimental data reported for those substances could indicate both autophagy activation and inhibition, requiring further investigation. Thus, the reviewed molecules were divided into two groups: having validated and non-validated autophagy modulatory effects. This review gives an analysis of the recent updates in the field and raises an important problem of standardization in the experimental design and data interpretation.

## 1. Introduction

### 1.1. Autophagy as a Biological Process

**Figure 1:**
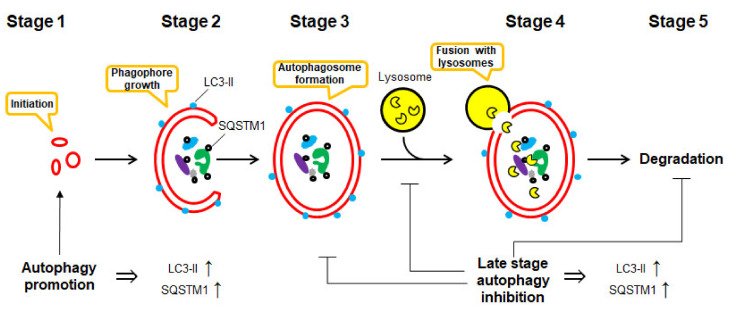
Autophagy flux.

Macroautophagy (here referred to as autophagy) is defined as a regulated mechanism of the degradation of unnecessary or dysfunctional cellular components [1]. This “self-eating” process may lead to the degradation—either selective or non-selective—of organelles and proteins by the lysosome system. It is the basic cellular catabolic “self-eating” process that leads to non-selective bulk degradation of organelles and proteins by the lysosome system [2]. It can result from cellular stress, e.g., starvation and exposure to toxins [3], and includes the formation of double-membrane vesicles (autophagosomes), which later fuse with lysosomes, leading to degradation and recycling of sequestered contents [2]. Biochemically, autophagy is an elegant and beautiful process, which consists of several critical stages, including (Figure 1):

**Stage** **1** induction: autophagy begins with the stimuli-initiation event (starvation, radiation, drug treatment, etc.).
**Stage** **2** phagophore formation: LC3-I converts to LC3-II via conjugation with phosphatidylethanolamine (PE). This process is called “lipidation”. “Phagophores” (or isolation membranes) are the double-membrane structures, which are growing in the cytosol.
**Stage** **3** autophagosome formation: phagophore engulfs bulk cytoplasm nonspecifically, including entire organelles; or it targets organelle cargos specifically, therefore forming vesicles called “autophagosomes”.
**Stage** **4** docking and fusion with lysosomes: the autophagosome fuses with a lysosome leading to the formation of “autolysosome”.
**Stage** **5** vesicle breakdown and degradation: finally, the sequestered material and organelles are degraded inside the autolysosome by lysosome proteases and recycled for the synthesis of ATP and various macromolecules such as proteins [4].

In the past few years, autophagy has received considerable attention, especially at the end of 2016, after the Nobel Prize in Physiology or Medicine “For the discoveries of mechanisms of autophagy” was awarded to Prof. Yoshinori Ohsumi [5]. However, it should be noted that in comparison with, e.g., apoptosis, autophagy is still a much less studied process [6].

Autophagy is a complex biological phenomenon. Over the last few years, several new and previously known proteins have been reported to be involved in this process [7,8]. Additionally, its crosstalk and interaction with basically every single biological process in different living organisms have been reported and investigated [9]. Thus, autophagy contributes to a number of physiological conditions, both normal and pathological, and plays a significant role in mammalian cells’ death and survival. Due to particular importance in oncology and different neurodegenerative disorders (e.g., Parkinson’s, Alzheimer’s disease and others), it has drawn great attention and has been closely investigated by scientists across the world. In cancer, autophagy has emerged as one of the high-potential therapeutic targets, which in some cases might be cancer-cell-specific and therefore represent a chance to reduce the side effect of the therapy [10]. This is why the idea of searching and developing compounds that can control and modulate this process is so attractive.

### 1.2. Blue-Print Autophagy

Remarkably, the chemical structures and variety of natural compounds found in marine organisms differ significantly from terrestrial plants and animals. Specific environmental conditions have been identified as the main reasons for this phenomenon. Moreover, many marine organisms produce a large variety of unique small molecules, which are often used to protect themselves in a highly competitive marine environment. A significant proportion of these compounds exhibit potent biological activity, targeting one or several specific biological processes and, therefore, could be used for the therapy of human diseases. Some of them are already applied in clinics. The first marine-derived compound that was later developed into a clinically used drug was spongothymidine [11,12,13]. This molecule was discovered in 1951 and later became a lead compound for the cytarabine development [14]. Nowadays, thirteen marine-derived drugs are used clinically, mainly as therapeutics for the treatment of cancer and cancer-related conditions [14,15,16,17,18]. Two of these drugs were approved in 2019 and at least one in 2020 (as of August 2020). Many more are in all different phases of testing within clinical trials, and a plethora of substances have already been preclinically tested in vitro and in vivo [19,20,21]. Thus, marine-derived compounds are of particular interest to biomedical scientists across the globe.

The term “Blue-print autophagy” was introduced by Ruocco and colleagues in 2016 when the very first review article on autophagy-modulating marine-derived compounds was published in Marine Drugs [22]. That article covered around 20 marine compounds, which were described to possess a relevant activity [22]. Since then, the field of autophagy has grown notably. In particular, many new compounds isolated from marine sources have been described as autophagy modulators (both activators and inhibitors) [23,24]. Some of these compounds are of special interest to scientists not only as biochemical tools but also as potential therapeutics for various pathological conditions. A good example of these molecules is new macrolides belonging to the famous family of autophagy inhibitors bafilomycins, which were isolated from the marine-derived strain of *Streptomyces* spp. [25]. According to the PubMed database (U.S. National Library of Medicine NIH), the annual number of the scientific papers related to “autophagy” has increased more than 50 times over the past 20 years [26]. Despite the relative specificity of the “blue-print autophagy” topic, this area is also continually growing along with the “mother” autophagy research field. Thus, a search Pubmed database indicates its 25-fold growth over the same period, i.e., from only one related manuscript/year published in 1999, to 25 manuscripts/year in 2019 (and 19 manuscripts have been already published by August of 2020) [27].

The current review is intended to not only give an overview of the compounds but also to critically analyze all the autophagy modulators isolated from marine organisms and reported within the beginning of 2016 to the end of August 2020. The source organism and molecular type of the bioactive molecules as well as an effect on autophagy and related molecular targets are reported. Particular attention was paid to the validation of the suggested autophagy-modulating effects. The molecules that were proven to be the true autophagy modulators were differentiated from those requiring further detailed examination before they could be assigned to the group of either activators or inhibitors. The selection criteria are described in the following chapter.

### 1.3. Challenges in Autophagy Monitoring and Results Interpretation

The autophagy flux seems to be quite straightforward. However, the first impression of this biochemical process’ relative simplicity is deceptive. As was mentioned in the previous chapter, a number of molecules play important roles at the different stages of this complex process [7,28]. Additionally, autophagy crosstalks with a great number of biophysiological pathways [9]. One of the most important and well-studied molecules involved in autophagy is LC3 (microtubule-associated proteins 1A/1B light chain 3) [29]. During autophagy, LC3-I gets lipidated and converts to LC3-II, which can easily be monitored using, e.g., Western blotting [28,29]. The latter got further integrated into the phagophore double membrane (which later becomes a part of autophagosome and then autolysosome membrane). LC3-II is an essential protein for the formation of these structures. Therefore, LC3-II is the most often used marker of autophagosomes and autolysosomes [28,29].

Another well-known and often used protein to monitor the autophagy flux is a ubiquitin-binding protein sequestosome 1 (SQSTM1, or p62). SQSTM1 is a cargo protein that targets other proteins and delivers them to autophagosomes for selective autophagy (via binding with autophagosomal membrane LC3-II protein) [28,30].

Both LC3-II and SQSTM1 are upregulated when autophagy is activated and are degraded in late autolysosomes [7]. Thus, many authors, while detecting elevated LC3-II and SQSTM1 levels in the stimulated (e.g., drug-treated) cells, make false conclusions on the autophagy-activatory effect of the investigated stimuli. However, the inhibition of autophagy at the late stage (e.g., at the step of autophagosome fusion with lysosomes or at the step of autolysosomes degradation) also results in elevated LC3-II and SQSTM1 levels due to the disruption of autophagic turnover (Figure 1) [7,28,30,31]. Thus, autophagy inhibition may be giving a very similar, if not identical, picture as to when autophagy is activated [7,30]. Moreover, inhibition of autophagy at the early stages, on the contrary, may result in the downregulation of LC3-II and SQSTM1 expressional levels [7,30]. Finally, in some models, a time-dependent investigation of LC3-II protein level under the autophagy-inducing conditions has revealed its upregulation at the early time points following a further decrease. [7,30]. Therefore, monitoring of only LC3-II and SQSTM1 levels is certainly not enough to distinguish between such distinct effects as activation and inhibition of autophagy, especially in the models where this effect results from drug treatment [7,28,30,31].

The second challenge that the scientists working in the field of autophagy modulators face is an effect on cellular viability. In other words, the question to be answered―“does a particular autophagy promoter activates pro-survival or cytotoxic autophagy?” (or “does a particular autophagy inhibitor suppresses pro-survival or cytotoxic autophagy?”). This information is of critical importance for the further clinical application of the compound, especially for drugs, which are investigated as possible medications for cancer or neurodegenerative disorders. In the cellular context, autophagy’s biological effect can be either cytoprotective, cytotoxic, cytostatic, or even non-protective (no effect on viability or the sensitivity of cells to the drugs) [6]. Indeed, initially, autophagy has been described as a cytoprotective mechanism, which helps cells to survive the stress [1,3]. Note, autophagy induced in cancer cells exposed to the cytotoxic chemotherapeutics often exhibits cytoprotective characteristics [3,32]. However, excessive autophagy may lead to cellular death and is classified as a “programmed cell death type II” (apoptosis is a “programmed cell death type I”) [3]. Moreover, several natural compounds, also derived from the marine sources, have been reported to induce the autophagic cell death [22].

Since autophagy is a relatively young and sometimes still controversial topic, some standardization of the research methods, approaches, and interpretation of the results were needed. Thus, Prof. Daniel J. Klionsky and colleagues developed and published the very first “Guidelines for the use and interpretation of assays for monitoring autophagy in higher eukaryotes” in 2008 [30]. Here, Klionsky et al. have collated and critically analyzed the methods of autophagy monitoring as well as gave a recommendation on the interpretation of the experimental results. These Guidelines were then updated in 2012 (2nd edition) [31] and 2016 (3rd edition) [7], and finally, the latest 4th edition is about to be published in 2020 [8,33]. These Guidelines may be referred to as the gold standard when planning and analyzing autophagy-modulating drugs’ activity. Thus, the current review is intended to critically analyze the data on the autophagy-related activity of the marine-derived compounds, which were reported in the scientific literature over the last four years (beginning of 2016–August 2020). Following the recommendation by Klionsky et al. [7,30,31], the activity (activation/inhibition) of the reported autophagy modulators was assumed to be validated if:

(a) two or more independent methods were used to prove the suggested effect on autophagy;
(b) these methods could clearly distinguish inhibition and activation of autophagy in the biological model used.

If at least one of these criteria is not met, the effect of the certain compound on autophagy, suggested by the authors of the corresponding article, was considered as non-validated (for a detailed description, please refer to [7,30,31]). Importantly, according to the Guidelines, there are no absolute criteria or a single universal assay to determine an autophagic status in every biological model [7,30,31]. In fact, an opposing activity has been observed depending on the cell type used and the stimuli examined (i.e., a type of the drug) [3]. Therefore, careful attention should always be paid to each specific case, and the biological backgrounds of the model and drug should always be considered [7].

## 2. Marine Compounds with a Validated Autophagy-Modulatory Effect

**Figure 2:**
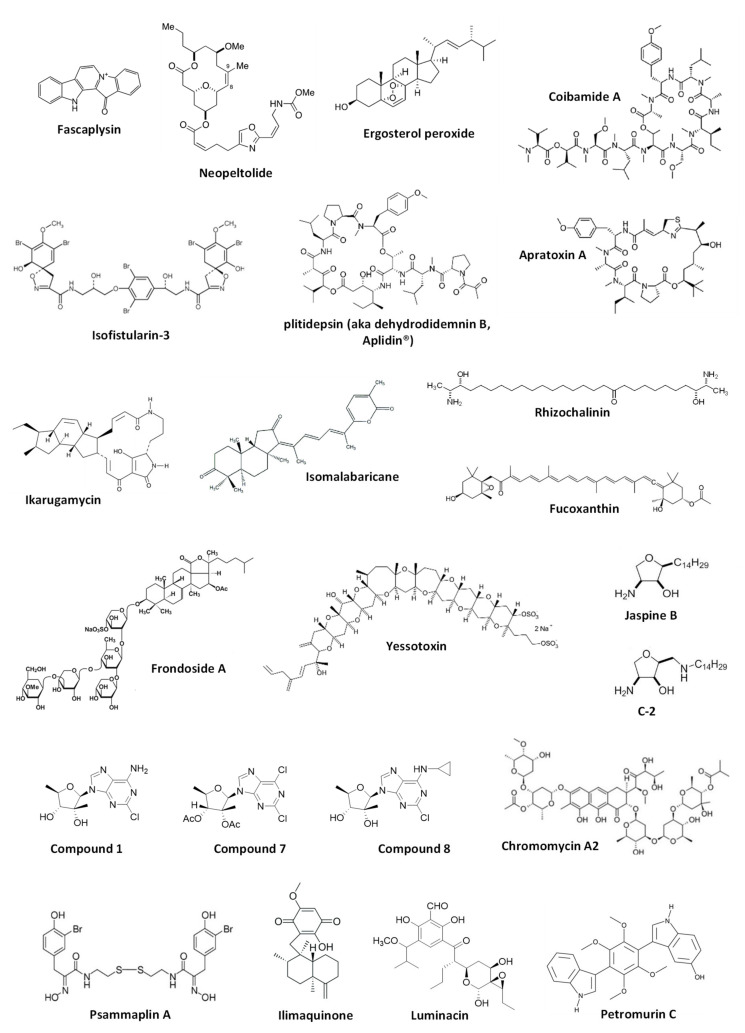
Marine-derived compounds with a validated autophagy-modulatory effect.

This chapter comprises marine-derived compounds reported to activate or inhibit autophagy in mammalian cells (Figure 2). For the compounds listed below, the effect has been validated according to the Guidelines by Klionsky et al. [7,8,30,31] and therefore, can be considered correct. The biological effect as well as autophagy-related targets, if any, are discussed. The data are summarized in Table 1.

### 2.1. Alkaloids

Marine alkaloid **fascaplysin** was initially isolated from the marine sponge *Fascaplysinopsis* sp. in 1988 [34] and later from other sponge species [35]. This compound exhibited anticancer activity in several human cancer models [35]. Fascaplysin was reported to be a potent inducer of cancer cell apoptosis mainly exerted via cyclin-dependent kinase 4 (CDK4) inhibition and suppression of angiogenesis [36,37]. Meng et al. have reported the activation of cytoprotective autophagy in human vascular endothelial cells (HUVEC) treated with this drug, which was identified as a resistance mechanism [38]. Thus, fascaplysin-induced autophagy could decrease a pro-apoptotic as well as anti-angiogenetic effects of this alkaloid. Increased expression of ROS (reactive oxygen species) and p8 protein was reported to be a factor related to the autophagy induction in HUVEC cells by fascaplysin [38]. Finally, the authors suggested that combined treatment with autophagy inhibitors could increase the anticancer activity of the alkaloid [38]. In this research, the authors used several independent methods to demonstrate an effect on autophagy; hence, the evidence of the autophagy-inducing activity of fascaplysin is quite convincing. In 2016, the group of Diederich reported a new activity [39] of the previously known brominated alkaloid **isofistularin-3**, which has been found in several marine sponges, including *Aplysina aerophoba*, *Verongia aerophoba* and others [40,41]. Thus, isofistularin-3 exerted anticancer activity via DNA demethylation and induced autophagy in Raji cells (Burkitt’s lymphoma) [39]. The effect of this brominated natural alkaloid on autophagy was confirmed using several methods, including combination with autophagy inhibitors. However, the authors did not investigate the role of this process in the isofistularin-3-induced cancer cell death [39].

### 2.2. Macrocyclic Molecules

**Coibamide A** and **apratoxin A** are cytotoxic lariat depsipeptides isolated from marine cyanobacteria *Leptolyngbya* sp. [42] and *Lyngbya majuscule* [43], correspondently. The group of Ishmael has reported coibamide A to inhibit the tumor growth in vitro and in vivo in glioblastoma models [44]. They have also shown both coibamide A and apratoxin A to inhibit angiogenesis via suppression of VEGFR2 expression and induce autophagy in human non-cancer HUVEC cells. The latest finding was validated by the combinational experiments with bafilmycin A1 [44]. The effect of the induced autophagy on HUVEC cell viability is still to be investigated in the future. Using the mouse embryonic fibroblasts (MEFs) model two years later, the same group has shown that ATG5 is required for the cytotoxic activity of these depsipeptides, suggesting induction of cytotoxic autophagy [45]. Additionally, it was postulated that induced autophagy is not triggered by acute ER stress [45].

Another well-representative example of the discovery of autophagy-modulating marine natural compounds is the research performed by the group of Gao and White, who have developed a nanotechnology-enabled high-throughput screening system for the identification of transcription factor EB (TFEB) activators [46]. TFEB is known to be activated in starvation conditions and ultimately promotes autophagy. Thus, the authors have screened 15,000 natural and synthetic compounds and identified, among others, **ikarugamycin** as a potent agonist of TFEB. Ikarugamycin is a natural macrocyclic antibiotic initially isolated from marine-derived bacteria *Streptomyces phaeochromogenes* [47]. This compound was able to increase the concentration of cytosolic Ca^2+^, which resulted in the consequent activation of CaMKKβ and AMPK pathway, inhibition of mTORC1, activation of TFEB, and ultimately in promotion of autophagy [46]. Hence, it was shown that ikarugamycin could activate autophagy in the HeLa cell model in vitro, ameliorate metabolic syndrome in the mice model and extend *C. elegans* lifespan in vivo via the same mechanism [46].

The cyclic depsipeptide **plitidepsin**, also known as **dehydrodidemnin B** or as the anticancer drug **Aplidin^®^**, was initially extracted from the ascidian *Aplidium albicans* [48]. Losada et al. have explored the effect of plitidepsin in HeLa cells [49]. The authors could show and prove plitidepsin to induce ER stress and simultaneously inhibit proteasome- and autophagy-mediated degradation of misfolded proteins [49]. This results in an unfolded protein response, stipulated by the alternative XBP1 splicing, the proteolytic processing of ATF6 as well as phosphorylation of JNK and eIF2α. Of note, distinctly from the most autophagy inhibitors, plitidepsin was able to inhibit autophagy at the early stages, preventing LC3-I lipidation to LC3-II. Moreover, the authors showed that this effect was not due to the inhibition of Vps34 (PIK3C3) or activation of mTOR. Thus, it was suggested that the autophagy-inhibitory effect was eventually due to the direct binding of plitidepsin to eEF1A2, which, therefore, could be one of the molecular targets of this drug [49]. Another interesting research by Fuwa and Sato reports an anticancer activity of the synthetic analog of marine macrolide neopeltolide (isolated from a deep-water sponge *Neopeltidae* sp. [50])—**8,9-dehydroneopeltolide** (**8,9-DNP**)—in human pancreatic cancer PANC-1 cells [51]. The authors have shown 8,9-DNP to inhibit cytoprotective autophagy by preventing lipidation of LC3B-I to LC3B-II under starvation conditions. Even though the drug target was not identified, the generated results suggested that autophagy was inhibited at its early stages and that 8,9-DNP does not interfere with cellular energy-sensing signals [51].

### 2.3. Triterpenes

An examination of the anticancer activity of marine triterpene glycoside **frondoside A**, isolated from sea cucumber *Cucumaria frondosa* [52] using in vitro and in vivo models of human drug-resistant prostate cancer cells by Dyshlovoy et al. has revealed the time-dependent accumulation of autophagosomes [53]. Further examination of LC3B-II accumulation dynamic and combinational experiments suggested the inhibition of pro-survival autophagy in prostate cancer cells by frondoside A. The investigation of the biological activity in human bladder cancer [54], as well as Burkitt’s lymphoma models in vitro [55] by the same group, revealed the similar signs of autophagy inhibition. At the same time, as the autophagy-modulatory effect of the same drug may significantly differ depending on the experimental model used [7,8,30,31], autophagy inhibition by frondoside A in bladder cancer as well as in Burkitt’s lymphoma is still to be validated. **Ergosterol peroxide** was isolated from the marine fungus *Phoma* sp. and was described to be cytotoxic to the human lung adenocarcinoma cells [56]. On the other hand, this natural compound could induce autophagy in these cells, which was revealed to be cytoprotective. This cellular process attenuated the anticancer effects of ergosterol peroxide and was associated with ROS induction as well as with regulation of ERK, JNK and p38 MAPK, and other proteins [56]. The group of Kong has reported an isomalabaricane triterpene secondary metabolite of marine sponge *Jaspis stellifera*, **stellettin B** [57], to induce autophagy in human non-small cell lung cancer [58]. The inhibition of PI3K/Akt/mTOR pathway via suppression of PI3K-p110 has been suggested as a mechanism of autophagy induction in these cells. The authors speculated this process to contribute to the cytotoxic activity of stellettin B [58]. However, even though the autophagy-inducing effect was clearly proved, additional experiments are required to clarify the suggested cytotoxic role of the stellettin B-induced autophagy.

### 2.4. Other Molecules

**Yessotoxin** (**YTX**) is a polyether molecule produced by the dinoflagellates *Protoceratium reticulatum* and *Gonyaulax grindleyi* [59,60]. YTX has been reported to have a broad spectrum of biological activity while the mechanism of action may vary significantly depending on concentration, time of exposure and the model used. In mammalian cells, its cytotoxic action was reported to be associated with ER and ribotoxic stress [61,62]. In 2016, Korsnes et al. reported activation of autophagy by YTX in mouse brain tumor BC3H1 cells (which also possess some properties of smooth muscle cells) [63]. The authors used several methods, including electron microscopy, IHC and co-treatment with inhibitors, to prove autophagy activation by this natural compound. They speculated on the presence of the potential cross-talk between autophagy initiated by the drug-induced ribotoxic stress and cell death pathways. However, neither the autophagy associated molecular target of YTX, nor the precise effect of the observed autophagy on the viability of the tumor cells was identified [63]. Of note, in 2014 yessotoxin was reported to induce ER-stress-associated cytotoxic autophagy in human glioma cells [61]. **Rhizochalinin** is a semi-synthetic aglycon of an unusual marine two-headed sphingolipid rhizochalin, which was initially isolated from the marine sponge *Rhizochalina incrustata* [64,65]. This molecule possesses a wide range of biological activities [66,67,68,69]. Dyshlovoy et al. have reported the inhibitory effect of the rhizochalinin on cytoprotective autophagy in human drug-resistant prostate cancer cells [70]. This effect was confirmed using several methods. The authors postulated that the inhibition of autophagy by this lipid compound contributes to its anticancer effects in vitro and in vivo. It was also confirmed, that inhibition of voltage-gated potassium channels is a direct cellular target of rhizochalinin [70], however, its potential crosstalk with an effect on autophagy is still to be investigated. Later, the same group has reported the synthesis of 18-hydroxy- and 18-aminoderivatives of rhizochalin and rhizochalinin, for which the same effect on LC3-B-I/II expression in the same model was shown [69]. In their former research, the authors, however, have not confirmed the suggested inhibitory effect of the compounds. Nonetheless, it is highly likely that the synthesized derivatives exert the same autophagy inhibitory effect as the original rhizochalinin molecule [69]. The group of Koumbis has synthesized the derivatives of **trachycladines**, the naturally occurred bioactive nucleosides that were initially found in the marine sponge *Trachycladus laevispirulifer* [71] and *Theonella* sp. [72]. The authors have shown the synthesized compounds **1**, **7** and **8** to bear anticancer properties and inhibit autophagy in human cervical carcinoma HeLa cells via inhibition of the fusion of autophagosome and lysosomes [73]. As this effect was demonstrated by several independent methods [73], the inhibitory effect trachycladines on autophagy may be considered verified [7,8,30,31].

Luminacins are secondary metabolites of the marine bacteria *Streptomyces* sp. [74]. Shin et al. have examined the anticancer effect of **luminacin** in vitro using the model of head and neck squamous cell carcinoma (HNSCC) as well as in vivo using the zebrafish model [75]. In this research, it was reported that luminacin induces autophagy, which was confirmed by several experiments with the compound alone as well as in combination with autophagy inhibitors. Simultaneously, the activation of p38 and JNK MAPK, as well as the inhibition of Akt, were observed. The authors postulated that the induced autophagy has a cytotoxic character and contributes to the compound-induced cell death [75]. However, this statement has not been validated experimentally and therefore, is still to be confirmed, especially as drug-induced autophagy induced in cancer cells may often have a pro-survival character [3,7,8,30,31]. **Fucoxanthin** was first isolated from the marine algae *Fucus* sp., *Dictyota* sp., and *Laminaria* sp. and later found in other Ochrophyta representatives, including brown algae (Phaeophyceae) and diatoms (Bacillariophyta) (reviewed in [76]). Feng at al. have shown that this marine algal carotenoid inhibits the proliferation of nasopharyngeal carcinoma (NPC) cells [77]. Their research also identified the induction of cytotoxic autophagy in NPC cells under the treatment, which was confirmed by the combined treatment with autophagy inhibitors [77]. This effect was ROS-mediated and no further molecular targets were identified. Another noteworthy report on fucoxanthin bioactivity was published by the group of Wang. The authors described its neuroprotective activity in the traumatic brain injury (TBI) model in vivo and ex vivo [78]. It was suggested that fucoxanthin executes its neuroprotective effect via the activation of Nrf2-ARE and Nrf2-autophagy pathways [78]. The conclusion on fucoxanthin-promoted autophagy in the model of TBI was made based on the formation of LC3 puncta, upregulation of Beclin-1 and LC3-II, and downregulation of p62. Whereas in Nrf2^-/-^ mice, these effects could not be observed [78]. It should be noted that even though the observed signals, especially in in vivo models, are most likely attributed to the autophagy induction, additional experiments (e.g., the combination with late-stage autophagy inhibitors) would be useful to verify the suggested pro-survival nature of autophagy induced. The research by Liao et al. describes an anticancer in vitro and in vivo effects of **phycocyanin**, a pigment-protein complex belonging to the light-harvesting phycobiliprotein family, in the pancreatic cancer model [79]. Using a number of methods, including siRNA silencing of autophagy-related genes, immunohistochemistry, and DQ-BSA quenching assay, and authors have proved that the mechanism of action of phycocyanin includes induction of both apoptosis and cytotoxic autophagy. Moreover, it was shown that the mechanism of action is represented as a complex cross-talk between MAPK (p38, JNK and ERK), Akt/mTOR/p70S6K, and NF-κB pathways [79].

A good example of research on a marine-derived compound as possible autophagy modulators is the investigation of **jaspine B** and its **2-alkylaminomethyl derivatives** performed by two different groups [80,81]. Jaspine B is an anhydrophytosphingosine that was first found in the marine sponge *Pachastrissa* sp. [82]. An extensive examination of the effect of this natural compound on gastric cancer cells was performed by Cingolani et al. [81]. The researchers have observed cytotoxic effects of jaspine B coupled with the cancer cell membrane vacuolization, which, however, could not be inhibited by the pan-caspase inhibitor zVAD nor by the autophagy inhibitor wortmannin. Moreover, the accumulated LC3-II-positive structures appeared to be the single-membrane vacuoles, and therefore, could not be identified as double-membrane autophagosomes. Consequently, the detected membrane vacuolization, which ultimately triggered a cytoplasmic disruption, was classified as a result of the autophagy-unrelated micropinocytosis [81]. The more recent research by the group of Jin, performed by Zhang et al., has reported a synthesis of several cytotoxic jaspine B derivatives and suggested these compounds to bear an autophagy-promoting activity in prostate cancer cells [83]. This speculation, however, requires further validation [83]. Nevertheless, in the following research by Yu et al. (Jin group), the authors have applied a number of diverse methods to prove the autophagy-inducing activity of the most promising jaspine B derivative called **C-2** in human bladder cancer cells [80]. Thus, the authors successfully demonstrated the importance of JNK and Nrf2 pathways for the C-2-induced autophagy, which seems to be cytoprotective, in bladder cancer cells. Finally, the anticancer and autophagy-inducing activity as well as the mode of action of C-2 were validated in vivo using the nude mice xenografts [80]. The rather contradictive results of these two high-quality pieces of research performed by Cingolani et al. [81] and Yu et al. [80] could be explained by the different models used, namely, gastric [77] and bladder cancer cells [80], as well as by the chemical difference in the original natural jaspine B [81] and its synthetic derivative C-2 [80].

Ratovitski has reported an evaluation of the autophagy-modulatory activity of **chromomycin A2** (from marine-derived bacteria *Streptomyces* sp. [84]), **psammaplin A** (from marine sponge *Psammaplysilla* sp. [85]), and **ilimaquinone** (from marine sponge *Hippospongia metachromia* [86]) in vitro using the models of human squamous cell carcinoma, glioblastoma, and colorectal carcinoma cells [87]. It was shown that these compounds exhibit cytotoxic activity in the cancer cells and induce autophagy, which seems to contribute to the observed anticancer effects. The induction of autophagy was validated using co-treatment with bafilomycin A1. It was also shown that the transcription of the autophagy-related genes is activated in the treated cells, and this process is regulated by the treatment-induced TP53 family members’ transcriptional activity [87]. Thus, the treatment with chromomycin A2, psammaplin A, and ilimaquinone induced the expression of TP53, TP63 and TP73 as well as phosphorylation of these proteins. At the same time, silencing of TP53, TP63 and TP73 expression resulted in the inhibition of the expression of several autophagy-related genes as well as in the reduction of the drug-induced expression of these genes [87]. He et al. have reported anticancer activity of **petromurin C** in the model of FLT3-ITD-positive AML in vitro using the cell lines MV4-11 and U937 as well as the toxicity of this compound in vivo in the zebrafish model [88]. Petromurin C is a secondary metabolite of a fungus *Aspergillus candidus* KUFA0062 (which was in turn isolated from the marine sponge *Epipolasis* sp. [89]). Initially, petromurin C was isolated from *Petromyces muricatus* [90]. The authors have detected the accumulation of autophagosome/autolysosome-like structures as well as LC3B-I/II in the treated cancer cells [88]. Combinational treatment with bafilomycin A1 could significantly increase the petromurin C-induced accumulation of LC3B-II suggesting the induction of autophagy by the second compound. On the contrary, the authors have reported bafilomycin A1 to significantly decrease the accumulation of vacuoles [88]. Being an inhibitor of the late autophagy stages, bafilomycin A1 usually leads to the accumulation of autophagosomes as a result of inhibition of their fusion to lysosomes (which, therefore, results in prevention of autolysosome formation and degradation) [91]. Thus, the nature of petromurin C-induced vacuoles and the effect of bafilomycin A1 on their formation should be further studied.

**Table 1:** Marine compounds with a validated autophagy-modulatory effect ^1^.

Name	Source Organism	Suggested Effect on Autophagy	Effect validated? ^1^	Target ^2^	Molecular Class	Model	Ref.
**Alkaloids**							
Fascaplysin	Marine sponge *Fascaplysinopsis* sp., and others	Activation of cytoprotective autophagy	Yes	p8 protein; ROS	Alkaloid	Vascular endothelial cells (HUVEC cells)	[38]
Isofistularin-3	Marine sponge *Aplysina aerophoba*	Activation	Yes	-	Alkaloid	Burkitt’s lymphoma (Raji cells)	[39]
**Macrocyclic molecules**							
Coibamide A	Marine cyanobacteria *Leptolyngbya* sp.	Activation	Yes	VEGFR2	Cyclic depsipeptide	Human umbilical vein endothelial cells (HUVEC)	[44]
		Activation of cytotoxic autophagy	Yes	-(was shown that ATG5 is required)		Mouse embryonic fibroblasts (MEF cells)	[45]
Apratoxin A	Marine cyanobacteria *Lyngbya majuscule*	Activation	Yes	VEGFR2	Cyclic depsipeptide	Human umbilical vein endothelial cells (HUVEC)	[44]
		Activation of cytotoxic autophagy	Yes	-(ATG5 is required, whereas acute ER stress is not important)		Mouse embryonic fibroblasts (MEF cells)	[45]
Ikarugamycin	Marine bacteria *Streptomyces phaeochromogenes*	Activation	Yes	ER; CaMKKβ and AMPK pathways; mTORC1; TFEB	Macrocyclic antibiotic	Cervical carcinoma in vitro (HeLa cells); metabolic syndrome in vivo (mice); lifespan in vivo (C. elegans)	[46]
Plitidepsin (aka dehydrodidemnin B; Aplidin^®^)	Ascidian *Aplidium albicans*	Inhibition	Yes	eEF1A2; ER stress	Cyclic depsipeptide	Cervical carcinoma (HeLa cells)	[49]
8,9-Dehydroneopeltolide (8,9-DNP)	Marine sponge *Neopeltidae* sp. (synthetic derivative)	Inhibition of cytoprotective autophagy (at early stages)	Yes	-	Macrolide	Pancreatic cancer (PANC-1 cells)	[51]
**Triterpenes**							
Frondoside A	Sea cucumber *Cucumaria frondosa*	Inhibition of cytoprotective autophagy	Yes	-	Triterpene glycoside	Prostate cancer	[53]
		Inhibition	No	-		Bladder cancer	[54]
		Inhibition	No	-		Burkitt’s lymphoma	[55]
Ergosterol peroxide	Marine fungus *Phoma* sp.	Activation of cytoprotective autophagy	Yes	ERK; JNK; p38; AKT; mTOR and others	Sterol	Lung adenocarcinoma cells (A549 cells)	[56]
Stellettin B	Marine sponge *Jaspis stellifera*	Activation	Yes	PI3K-p110; PI3K/Akt/mTOR pathway	Isomalabaricane triterpene	Non-small cell lung cancer (A549 cells)	[58]
**Another molecules**							
Yessotoxin	Dinoflagellates *Protoceratium reticulatum* and *Gonyaulax* *grindleyi*	Activation	Yes	ER- and ribotoxic stress	Polyether	Mouse brain tumor (BC3H1 cells)	[63]
		Activation of cytotoxic autophagy	Yes	mTOR; BNIP3		Glioma (SF295, SF539, and SNB75 cells)	[61]
Rhizochalinin and the derivatives	Marine sponge *Rhizochalina incrustata* (semisynthetic derivative)	Inhibition	Yes	-	Lipid	Prostate cancer (PC-3 cells)	[69,70]
Trachycladines derivatives (Compound 1, 7 and 8)	Marine sponges *Trachycladus laevispirulifer* and *Theonella* sp. (synthetic analogue)	Inhibition	Yes	-	Nucleoside	Cervical carcinoma (HeLa cells)	[73]
Luminacin	Marine bacteria *Streptomyces* sp.	Activation	Yes	p38; JNK; Akt	Secondary metabolite	Head and neck squamous cell carcinoma (HNSCC); zebrafish	[75]
Fucoxanthin	Various brown algae and diatoms	Activation of cytotoxic autophagy	Yes	ROS	Carotenoid	Nasopharyngeal carcinoma	[77]
		Activation of cytoprotective autophagy	No	Nrf2 signaling		In vivo traumatic brain injury; primary cultured neuron	[78]
Phycocyanin	Cyanobacteria (*Arthrospira* sp. aka Spirulina)	Activation of cytotoxic autophagy	Yes	MAPK, Akt/mTOR/p70S6K and NF-κB pathways	Pigment-protein complex	Pancreatic cancer cells (PANC-1 cells)	[79]
Jaspine B	Marine sponge *Pachastrissa* sp.	No effect (autophagy-unrelated vacuolization of cytoplasm)	Yes	-	Cyclic anhydrophytosphingosine	Gastric Cancer (HGC-27 cells)	[81]
C-2 (2-alkylaminomethyl derivatives of jaspine B)	Marine sponge *Pachastrissa* sp. (synthetic analogue)	Activation of cytoprotective autophagy	Yes	JNK; Nrf2 pathway	Cyclic anhydrophytosphingosine, 2-alkylaminomethyl derivative	Bladder cancer (BIU87, 5637 and EJ cells)	[80,83]
Cromomycin A2	Marine bacterium *Streptomyces* sp.	Activation	Yes	TP53 family members (TP53, TP63 and TP73)	Anthraquinone antibiotic glycoside	Squamous cell carcinoma (SCC-11 cells)	[87]
Psammaplin A	Marine sponge *Psammaplysilla* sp.				Bromotyrosine-cystamine conjugate	Glioblastoma (U87-MG cells)	
Ilimaquinone	Marine sponge *Hippospongia metachromia*				Prenylquinone; monohydroxy-1,4-benzoquinones	Colon colorectal cancer (RKO cells)	
Petromurin C	Marine fungus *Aspergillus candidus* KUFA0062, and others	Activation	Yes	Mitochondrial stress; Mcl-1	bis-Indolyl benzenoid	Acute myeloid leukemia (AML) (MV4-11 and U937 cells)	[88]

^1^ Effect validated using several independent methods (according to the Guidelines by Klionsky at al. [7,8,30,31]); ^2^ Direct or indirect target of the compound suggested to be related to the effect on autophagy.

## 3. Marine Compounds with a Non-Validated Autophagy-Modulatory Effect

**Figure 3:**
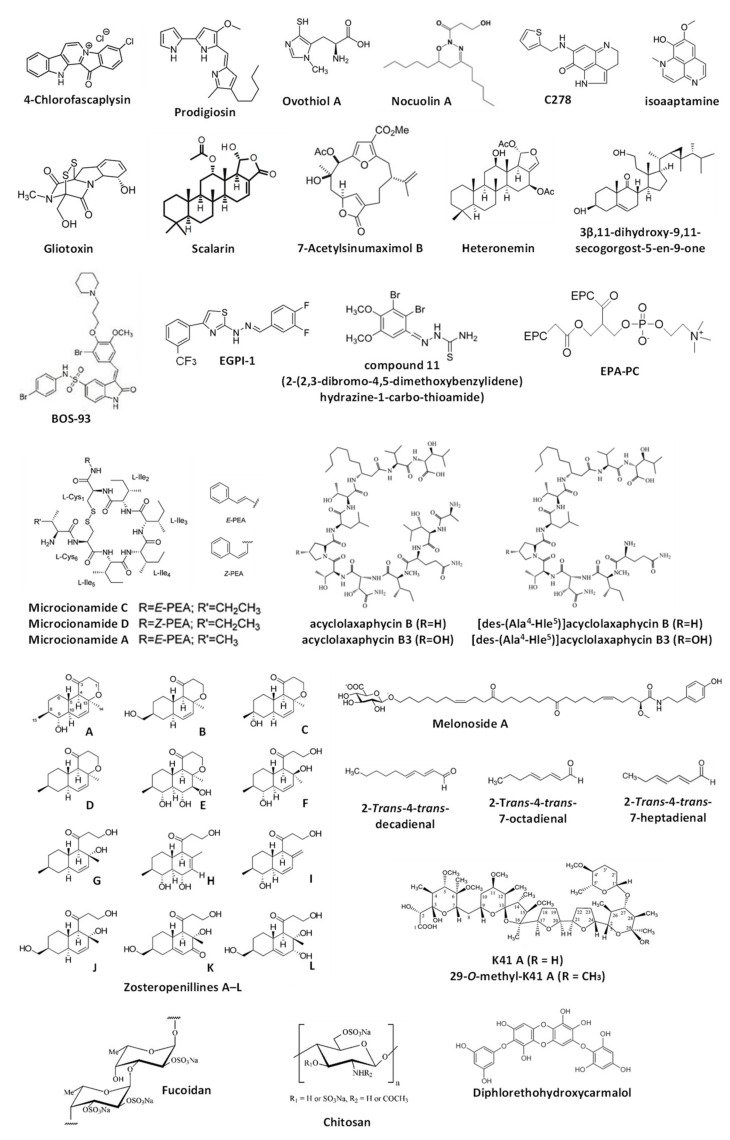
Marine-derived compounds with non-validated autophagy-modulatory effect.

The current chapter overviews the molecules isolated from marine organisms that were reported to modulate autophagy but, according to the Guidelines by Klionsky et al., not yet validated as such [7,8,30,31] (Figure 3). Note, for all these compounds, the alterations of one or several autophagy markers are shown; therefore, these substances can be assumed as autophagy modulators. However, as the detected signals may be assigned to both promoted and suppressed autophagy, the suggested effect (i.e., either autophagy activation or inhibition) cannot be assumed as valid. For the compounds listed below, further experiments should be considered to clarify their effects on autophagy in certain models. The data are summarized in Table 2.

### 3.1. Alkaloids

In the previous chapter, the autophagy-inducing activity of the marine alkaloid fascaplysin was reported. Another piece of research by Sharma et al. describes the activity of **4-chlorofascaplysin** in human breast cancer cells [92]. The compound could inhibit the PI3K/Akt/mTOR pathway in MDA-MB-231 cells. The authors have suggested induction of cytotoxic autophagy by the drug as an upregulated acridine orange staining as well as elevated LC3-II level were detected in the treated cells [92]. This speculation requires further confirmation since the observed signs could also be attributed to the autophagy inhibition. Moreover, the confirmation of the cytotoxic nature of the induced autophagy, if it exists, requires additional evidence.

**Prodigiosin**, a cytotoxic alkaloid with antimicrobial and anticancer activities, has been initially isolated from the red pigment of Gram-negative marine bacteria *Serratia marcescens* [93] and later from the marine Gram-negative γ-proteobacteria *Vibrio* sp. as well as other species [94,95]. Cheng at al. have described an anticancer activity of prodigiosin in human glioblastoma (GBM) cells and suggested this compound to activate autophagy in GBM cells via the activation of JNK and simultaneous inhibition of AKT/mTOR pathways, which was probably related to the prodigiosin-induced ER stress [96]. As the autophagy inhibitor 3-methyladenine was able to suppress the drug-induced cell death, the authors concluded that the investigated marine-derived compound induces cytotoxic autophagy in cancer cells [96]. At the same time, the observed upregulation of LC3-II and accumulation of LC3-II-positive organelles, which authors have provided as evidence for autophagy induction, may be observed during the autophagy inhibition too [7,8,30,31]. Therefore, additional experiments may be useful to confirm the autophagy-inducing activity of prodigiosin. The same research group has examined the anticancer activity of prodigiosin in the model of oral squamous cell carcinoma in vitro [97]. The authors reported that this compound may induce cytotoxic autophagy in these cells via inhibition of mTOR, Akt, and rpS6 phosphorylation, probably due to the cytotoxicity-induced degradation of the phosphor-forms of these proteins [97]. Moreover, the effect of autophagy inhibitor 3-MA on the cytotoxic activity of the alkaloid was controversial and dependent on its concentration. As no additional validation experiments were performed, and the observed results could indicate both induction and inhibition of autophagy (which could be either cytotoxic or cytoprotective), further examination is needed to confirm the suggested effects of prodigiosin on autophagy.

**Ovothiol A** is a 1-N-methyl-4-mercaptohistidine that has been isolated from eggs of the sea urchin *Paracentrotus lividus* as well as from other marine invertebrates (sea stars, cephalopods) [98]. Brancaccio et al. have reported this compound to inhibit the activity of γ-glutamyl transpeptidase in human cancer cells [99]. Based on the observed LC3-II upregulation and inhibitory effect of such autophagy inhibitors bafilomycin A1 and 3-methyladenin on the cytotoxic activity of ovothiol A, the authors concluded that this marine compound activates cytotoxic autophagy [99]. However, as the accumulation of LC3-II may also be observed during the inhibition of autophagy at the late stages, the conclusions made in this manuscript await additional confirmations. The group of Urbatzka reported an antiproliferative effect of marine natural oxadiazine **nocuolin A** (isolated from cyanobacteria *Nodularia* sp. LEGE 06071) on human colon cancer HCT116 cells [100]. Among others, the authors have reported mitochondria targeting, which results in mitochondrial oxidative phosphorylation (OxPhos) impairment. Additionally, the observed accumulation of LC3B-II and of autophagosome-like structures led to the preliminary conclusion on nocuolin A-induced autophagy induction. However, this should be confirmed by further experiments that will exclude the possible autophagy-inhibitory effect of nocuolin A, stipulated by the same signs. Natural pyrroloiminoquinone alkaloids **makaluvamines** were found in different marine sponges, i.e., *Zyzzya* cf. *marsailis*, *Histodermella* sp., *Zyzzya fuliginosa*, *Smenospongia aurea* and others (reviewed in [101]). Cowan et al. have reported the cytotoxic activity of synthetic makaluvamine derivative **C278** in non-melanoma skin cancer SCC13 cells [102]. Moreover, the authors have reported C278 to induce the expression of Beclin-1 [102]. Based on this result, the authors have suggested this alkaloid to induce autophagy in cancer cells. As no other experiments have been performed, this suggestion awaits thorough verification. Wu et al. have studied the activity of **isoaaptamine** in human breast cancer T-47D cells [103]. This and related alkaloids were isolated from marine sponges mainly belonging to *Aaptos* genus [104,105,106]. The compound induces ER stress, ROS production and disruption of mitochondrial membrane potential (Δψ_m_) [103]. Based on observed regulation of several autophagy-related proteins and accumulation of autophagosome-like structures, the authors suggested the activation of autophagy in the treated cells [103]. Thus, an upregulation of LC3B-II and p62/SQSTM1, as well as downregulation of total mTOR, was detected. At the same time, the authors did not report an alteration of phospho-mTOR. As these signs may be attributed to both activation and inhibition of late stages of autophagy, additional experiments would be required to clarify the effect of isoaaptamine on this basic cellular process. Park and colleagues have investigated the effect of **gliotoxin**, a secondary metabolite of marine fungus *Aspergillus fumigatus* (initially isolated from *Trichoderrna lignosum* [107]), in combination with paclitaxel [108]. The authors used paclitaxel-resistant ovarian cancer cells, which were consequently treated with gliotoxin and paclitaxel (GTX→PTX). This treatment led to the activation of DAPK1/TAp63 signaling [108]. Furthermore, the upregulation of LC3B-I/II levels together with increased apoptosis stipulated by the loss of mitochondria membrane potential (Δψ_m_) were detected in both CaOV3/PTX_R and SKOV3/PTX_R cells exposed to GTX→PTX. Of note, these effects could be abolished by the pre-treatment with autophagy inhibitor 3-methyladenine. As a result, the authors have suggested that gliotoxin may enhance the cytotoxic autophagy in ovarian cancer cells. However, the speculation on autophagy activation by gliotoxin is still to be validated.

### 3.2. Terpenes and Similar Compounds

**Scalarin** is a bioactive metabolite initially isolated from the marine sponge *Spongia nitens* [109] and later from the sponge *Euryspongia* cf. *rosea* [110]. Guznam et al. have shown this compound to be active in human pancreatic cancer cells and able to inhibit the Receptor for Advanced Glycation End products (RAGE) [110]. The authors suggested that this effect leads to autophagy inhibition, as an accumulation of LC3-II in the scalarin-treated cells was detected. As no further evidence is presented, the experiments to validate the suggested effect are necessary. **7-acetylsinumaximol B** is a cembranoid (belonging to diterpenes) that was isolated by Tsai et al. from the marine cultured soft coral *Sinularia sandensis* in 2015 [111]. Later, the same group examined the anti-cancer activity of this natural compound in vitro in the human gastric carcinoma model and reported the induction of apoptosis and autophagy in NCI-N87 cells [112]. The suggested mechanism was reported as the induction of mitochondria dysfunction and activation of the PERK/eIF2/ATF4/CHOP signaling [112]. However, the conclusion about autophagy induction was made solely based on the observed increased expression of LC3-I and LC3-II along with other proteins belonging to the Atg family. Therefore, the effect on autophagy (activation vs. inhibition) cannot be considered validated.

Lee et al. have studied the effect of sesterterpenoid metabolite **heteronemin**, isolated from marine sponge *Hyrtios* sp. [113], in human prostate cancer LNCaP cells [114]. Apart from the anticancer activity in vitro and in vivo, which was related to oxidative stress, ER-stress, topoisomerase II and Hsp90 inhibition, the researchers observed the upregulation of cellular LC3B-II under the treatment and suggested activation of cytoprotective autophagy by the drug [114]. Even though the co-treatment with autophagy inhibitors 3-methyladenine and chloroquine increased the cytotoxic effects of the heteronemin in the cells, future additional experiments are essential to confirm the autophagy-inducing activity of the studied sesterterpenoid. The group of Bai and Sheu reported the activation of PPARγ and promotion of autophagy in human breast cancer MCF-7 cells treated with **3β,11-dihydroxy-9,11-secogorgost-5-en-9-one** [115], a stetol isolated from soft coral *Klyxum flaccidum* [116]. The authors showed a time-dependent upregulation of LC3B-II and p62/SQSTM1 in the treated cells (which could be inhibited by co-treatment with 3-methyladenine), accumulation of autophagosome-like structures, and a pro-survival effect of 3-methyladenine when applied together with the investigated stetol compound [115]. Despite the well-planned research, it should be noted that the detected signals may be attributed to either induction or inhibition of autophagy at its late stages. Consequently, additional experiments are required to clarify the effect of 3β,11-dihydroxy-9,11-secogorgost-5-en-9-one.

### 3.3. Bromphenols

Bromophenols are a small unique group of compounds mainly found in marine algae. They have also been detected in marine fungi, sponges, ascidians, and bryozoans. They possess various biological activities including the anti-diabetic, anticancer, antioxidant, antimicrobial, and others (reviewed in [117]). The group of Shi have designed and synthesized the novel bromophenol−thiosemicarbazone hybrid molecules, and shown its potent activity as selective PARP-1 inhibitors [118]. The authors indicated one of the synthesized compounds, **2-(2,3-dibromo-4,5-dimethoxybenzylidene)hydrazine-1-carbo-thioamide** (**compound 11**), to induce autophagy in human ovarian cancer SK-OV-3 cells. Thus, the authors showed an accumulation of autophagosomes in the drug-treated cells using several appropriate methods [118]. However, the elevated number of autophagosomes/autolysosomes may indicate both the activation and inhibition of autophagy depending on the stimuli and model used [7,8,30,31]. For this reason, further elucidation would be required to confirm the suggestion [7,8,30,31]. The same group has also reported the design and synthesis of another bromophenol–thiazolylhydrazone hybrid molecule, which could inhibit the interaction of translation initiation factors eIF4E and eIF4G, upregulate ROS as well as inhibit the mitochondrial function via mTOR/4EBP1 pathway [119]. For the most promising molecule, namely **EGPI-1** (where, in fact, all the bromine atoms were substituted), the authors reported an elevated level of LC-II along with LC3-positive structures in the lung carcinoma A549 cells exposed to this drug. Therefore, the activation of autophagy by EGPI-1 was postulated. At the same time, as has already been mentioned above, additional experiments would be required to prove that the activation of autophagy is indeed taking place. One more piece of research from the same authors reports the synthesis of the **BOS-93** (**3-(3-bromo-5-methoxy-4-(3-(piperidin-1-yl)propoxy)benzylidene)-N-(4-bromophenyl)-2-oxoindoline-5-sulfonamide**), a novel bromophenol derivative [120]. Its anticancer activity was tested in vitro and in vivo in human lung cancer A549 cells [121]. The authors have observed an accumulation of LC3-positive structures in the BOS-93-treated cancer cells as well as LC3-II accumulation, which could be inhibited by co-treatment with 3-methylademine. Based on this, the authors suggested BOS-93 to induce autophagy of the cells [121]. In line with this, the inactivation of PI3K/Akt/mTOR pathway was observed [121]. It is rather likely that BOS-93, being a cytotoxic compound, induces the cytoprotective autophagy in cancer cells. However, it should be noted that similar signs (the accumulation of LC3-positive formations, which could be suppressed by the early-stage autophagy inhibitors) can also be observed under the autophagy-inhibitory conditions. Therefore, additional experiments would be of use to confirm the suggestions of Guo et al.

### 3.4. Peptides

The two new cyclic cystine-bridged peptides, namely, **microcionamides C** and **D**, and the previously known **microcionamide A** were isolated from the marine sponge *Clathria basilana* by Mokhlesi et al. [122]. The compounds bear an anticancer activity and similar to another macrolide bafilomycin A1 induce autophagosome accumulation in murine embryonic fibroblast (MEF) cells under starvation conditions in a flow-cytometry based experiment. Based on this, an autophagy-inhibitory activity has been suggested for these cyclic peptide molecules. On the other hand, the observed signs could result from both activation and inhibition of autophagy and therefore, require further detailed investigation [7,8,30,31]. The group of Auvray reported an anticancer activity of the peptides **K092A** (NFDTDEQALEDVFSKYG) and **K092B** (EAPPEAAEEDEW) [123] isolated from the dogfish *Scyliorhinus canicula* L. and pE-K092D—a pyroglutamate-modified peptide derived from *S. canicula* peptide K092D (QLTPEALADEEEMNALAAR) [124]. These molecules were active in human prostate cancer cells [123,124]. Thus, the treatment of the prostate cancer MDA-PCa-2b cells resulted in the decreased red fluorescence of the cells when stained with acridine orange. Based on this, the authors have concluded that the peptides inhibit autophagy in the cancer cells [123,124]. However, this suggestion requires thorough verification, as acridine orange stains acidic organelles (e.g., lysosomes, autolysosomes) and does not specifically stain autophagosomes [7,8,30,31].

Alvariño et al. have reported the isolation of four acyclic peptides—**acyclolaxaphycin B** and **acyclolaxaphycin B3** (originally found in the marine cyanobacteria *Anabaena torulosa* [125]), as well as **[des-(Ala^4^-Hle^5^)]acyclolaxaphycin B** and **[des-(Ala^4^-Hle^5^)]acyclolaxaphycin B3**—from herbivorous gastropod *Stylocheilus striatus* [126]. The authors examined their activity in human neuroblastoma SH-SY5Y cells. It was shown that these acyclic peptides initiate the activation of AMPK, expression of Beclin-1, conversion of LC3-I into LC3-II, as well as the downregulation of p62, inactivation of mTOR, and partial inactivation of p70S6. Thus, the authors suggested that these four acyclic peptides may activate autophagy in human cancer cells. As no further investigation has been performed, this speculation awaits additional experiments. The group of Nam has examined the activity of the peptide **PYP15** (DPKGKQQAIHVAPSF) isolated from the alga *Pyropia yezoensis* [127] using the mouse skeletal muscle atrophy model (C2C12 cells) [128]. Previously, it was shown that the extract of this alga is capable of inhibition of dexamethasone-induced lipidation of LC3B-I to LC3B-II in the same model (see Section 4) [129]. The authors reported a similar effect of PYP15 on LC3B-I/II and on cathepsin L expression [128]. As no other experiments have been performed in both studies, additional data confirming the autophagy-inhibitory activity of PYP15 are needed.

### 3.5. Lipids

Eicosapentaenoic acid (EPA) is one of the natural products commonly found in edible marine organisms, such as antarctic krill, sea cucumber, and fish oil [130]. It primarily exists in phosphatidylcholine-conjugated forms [130]. Wen et al. have examined an effect of **eicosapentaenoic acid-enriched phosphatidylcholine** (**EPA-PC**) on Aβ1-42-induced neurotoxicity in vivo using the Alzheimer’s disease rat model [131]. The authors suggested that EPA-PC enhances autophagy in neuronal cells, therefore, attenuating Aβ1-42-induced neurotoxicity. This conclusion was made solely based on the results of the Western blotting-based examination of autophagy-related protein expression in the rat hippocampus and, therefore, awaits further experimental validation [131]. However, it should be noted that earlier studies have reported the autophagy inducing activity of EPA in several human cancer and non-cancer cells, which was, in the majority of cases, reported to be cytoprotective [132,133,134,135]. In 2016, Guzii et al. announced the isolation of an ω-glycosylated fatty acid amide **melonoside A** from the marine sponge *Melonanchora kobjakovae* [136]. The authors reported the induction of autophagy in human cisplatin-resistant germinal tumor (GCT) NCCIT-R cells. This conclusion was made based on the observed downregulation of LC3B-II and SQSTM1/p62 proteins in the treated cells [136], which often reflects the acceleration of the autophagosome turnover, i.e., increased autophagy [7,8,30,31]. However, further experiments are expected to clarify the effect of melonoside A on autophagy in mammalian cells. Galasso and colleagues have analyzed the effect of polyunsaturated aldehydes (PUAs), produced by different diatoms (reviewed in [137,138]), on the activation of several autophagy-related genes in the embryos of the sea urchin *Paracentrotus lividus*, as well as human lung cancer A549 cells [139]. Thus, the effects of **2-*trans*-4-*trans*-decadienal**, **2-*trans*-4-*trans*-7-octadienal**, and **2-*trans*-4-*trans*-7-heptadienal** were examined [139]. The authors identified the upregulation of the expression of three autophagy-related genes, namely ULK1/2, ULK3, and PINK, in both models under the treatment. However, this upregulation appeared to be significant only in the cells treated with 2-*trans*-4-*trans*-7-heptadienal [139]. The precise effect of these compounds on autophagy in current as well as in other models is to be further clarified.

### 3.6. Lectins

Li et al. have inserted the **expression cassette the *Ulva pertusa* lectin 1** (**UPL1**) in the adenovirus genome, and delivered it to the liver cancer BEL-7404 and Huh7 cells via infection [140]. Consequently, an expressed UPL1 affected several signaling pathways in the cells, including autophagy. It was observed that the exogenous UPL1 could inhibit Beclin1 and induce LC3-II expression in the cells; therefore, the authors suggested UPL1 for the enhancement of starvation-induced autophagy [140]. This speculation, however, requires further examination as no extra experiments have been performed. Do Nascimento-Neto et al. have examined an anticancer activity of **halilectin-3**, a lectin isolated from the marine sponge *Haliclona caerulea* [141], in human breast cancer MCF7 cells [142]. Among others, they have also reported the halilectin-3-induced accumulation of the acidic vesicles (presumably, autophagosomes) and LC3-II, and thus suggested the activation of autophagy in MCF7 cells [142]. Yet, as the observed signs may also be assigned to autophagy inhibition, the conclusion made by the authors requires further validation [7,8,30,31].

### 3.7. Polysaccharides

It has been reported that **3,6-*O*-sulfated chitosan**, which was derived from marine shrimp shells, is able to inhibit human papillomavirus (HPV) infection of HeLa cells via inhibition of PI3K/Akt/mTOR pathway [143]. Thus, Gao et al. suggested that 3,6-*O*-sulfated chitosan may activate autophagy (as some autophagy activators act via the same mechanism), which also contributes to the HPV infection suppression. However, no further experiments were performed. Hence, this speculation cannot be assumed to be validated [143]. **Fucoidans** are sulfated polysaccharides from brown algae containing mainly α-L-fucopyranose residues [144,145]. The group of Liu has investigated the hepatoprotective activity of this natural polymer isolated from *Fucus vesiculosus* in the in vivo model of CCl_4_- and bile duct ligation (BDL)-induced liver fibrosis [146]. The authors postulated that fucoidan executes its effect via TGF-β1/Smad pathway-mediated inhibition of cytotoxic autophagy and extracellular matrix formation. This conclusion was made based on the examination of LC3-I/II, SQSTM/p62 and Beclin-1 levels in the mice liver tissue [146]. Depending on the stimuli and model, the reported alteration of the protein levels could be attributed to both induction and inhibition of autophagy. Therefore, further experiments may be of use to clarify the observed modulatory effect of fucoidan on autophagy in the liver cells as well as the type of affected autophagy (cytoprotective/cytotoxic).

### 3.8. Other Metabolites

Khan et al. have reported the isolation of three polyether antibiotics from the marine actinobacterium *Streptomyces cacao*, namely, **arenaric acid**, **K41 A**, and **29-*O*-methyl-K41 A** [147]. These compounds possess anticancer properties which were demonstrated using human cervical carcinoma (HeLa), human prostate cancer (PC-3), human lung adenocarcinoma (A549), and human colorectal adenocarcinoma (CaCo-2) cells [147]. The effect of two of the isolated compounds on autophagy in cancer cell lines was also examined. The authors postulated that the isolated polyether antibiotics can inhibit autophagy. At the same time, several contradictions were observed and reported in this research article, in particular, the treatment-induced degradation of GFP-LC3 [7,8,30,31]. This phenomenon may be a sign of the drug-promoted autophagy, or (taking in account the downregulation of other proteins in the cells exposed to the cytotoxic drug concentrations) an unspecific effect of the cell death-related processes [7,8,30,31]. Therefore, further experiments resolving the observed contradictions are needed. Afiyatullov, Leshchenko et al. have reported the isolation of new polyketides **zosteropenillines A–L** from the marine-derived fungus *Penicillium thomii* [148]. The compounds were reported to be non-cytotoxic towards cancer cells, however, based on the observed upregulated level of SQSTM1/p62 in the treated cells, the authors suggested that the compounds are capable of autophagy inhibition [148]. As no other experiments have been performed, this suggestion awaits further validation. The group of Hyun has shown the **phlorotannin diphlorethohydroxycarmalol** (**DPHC**), isolated from edible seaweed *Ishige okamurae* [149], to protect skin cells from the fine particulate matter (PM)-induced damage caused by the PM-mediated ROS generation, DNA damage, and ER-stress [150]. This research was performed using a human non-cancer keratinocyte HaCaT cell line. Additionally, it was shown that DPHC can reduce PM-induced red acridine orange staining and LC3B-II level in the cells. Therefore, the authors suggested that DPHC inhibits PM-induced autophagy, which was probably due to the antioxidant activity of this compound, leading to a general reduction of the ROS-related cellular stress [150]. However, these statements await further examination.

**Table 2:** Marine compounds with non-validated autophagy-modulatory effect ^1^.

Name	Source Organism	Suggested Effect on Autophagy	Effect Validated? ^1^	Target ^2^	Molecular Class	Model	Ref.
**Alkaloids**							
4-Chlorofascaplysin	Marine sponge *Fascaplysinopsis* sp., and others (synthetic derivatives)	Activation	No	PI3K/Akt/mTOR	Alkaloid	Breast cancer (MDA-MB-231 cells)	[92]
Prodigiosin	Marine bacteria *Vibrio* sp.	Activation	No	JNK; AKT/mTOR; CHOP; ER stress	Alkaloid	Glioblastoma (GBM) (U87MG and GBM8401 cells)	[96]
	Bacteria *Serratia marcescens*	Activation of cytotoxic autophagy	No	mTOR, Akt, and rpS6		Oral squamous carcinoma (SAS and OECM1 cells)	[97]
Ovothiol A	Sea urchin *Paracentrotus lividus*	Activation	No	γ-Glutamyl transpeptidase (GGT)	Alkaloid	Leukemia (HG3 cells)	[99]
Nocuolin A	Cyanobacteria *Nodularia* sp. LEGE 06071	Activation	No	Mitochondria; Oxidative phosphorylation	Oxadiazine alkaloid	Colon cancer (HCT116 cells)	[100]
C278 (synthetic analog of makaluvamines)	Marine sponges *Zyzzya* spp. and others (synthetic derivative)	Activation	No	-	Pyrroloiminoquinone alkaloid	Non-melanoma skin cancer (SCC13 cells)	[102]
Isoaaptamine	Marine sponges *Aaptos* spp. and others	Activation	No	mTOR; ER stress; ROS; MMP	Alkaloid	Breast cancer (T-47D cells)	[103]
Gliotoxin	Marine fungus *Aspergillus fumigatus*	Activation of cytotoxic autophagy	No	DAPK1/TAp63 signaling	Alkaloid	Paclitaxel-resistant ovarian cancer (CaOV3/PTX_R and SKOV3/PTX_R cells)	[108]
**Terpenes and similar compounds**							
Scalarin	Marine sponge *Euryspongia* cf. *rosea* and others	Inhibition	No	Receptor for advanced glycation end products (RAGE)	Sesterterpene	Pancreatic cancer (PANC-1 and MIA PaCa-2 cells)	[110]
7-Acetylsinumaximol B	Soft coral *Sinularia sandensis*	Activation	No	Mitochondria dysfunction; PERK/eIF2/ATF4/CHOP signaling	Diterpene	Gastric cancer (NCI-N87 cells)	[112]
Heteronemin	Marine sponge *Hyrtios* sp.	Activation of cytoprotective autophagy	No	Oxidative and ER stress	Sesterterpenoid	Prostate cancer (LNCaP cells)	[114]
3β,11-Dihydroxy-9,11-secogorgost-5-en-9-one	Soft coral *Klyxum flaccidum*	Activation of cytotoxic autophagy	No	PPARγ; ROS	Stetol	Breast cancer (MCF-7 cells)	[115]
**Bromophenols**							
Bromophenol derivative (compound 11)	Various marine algae (synthetic analogue)	Activation	No	-	Bromophenol-thiosemicarbazone hybrid	Ovarian cancer (SK-OV-3 cells)	[118]
EGPI-1	Various marine algae (synthetic analogue)	Activation	No	eIF4E/eIF4G; mTOR/4EBP1 pathway; ROS	Bromophenol-thiosemicarbazone hybrid	Lung carcinoma (A549 cells)	[119]
BOS-93	Various marine algae (synthetic analogue)	Activation	No	PI3K/Akt/mTOR pathway; MAPK	Bromophenol derivative	Lung carcinoma (A549 cells)	[121]
**Peptides**							
Microcionamide A	Marine sponge *Clathria basilana*	Inhibition	No	-	Cyclic peptide	Murine embryonic fibroblasts (MEF cells)	[122]
Microcionamide C							
Microcionamide D							
K092A and K092B	Dogfish *Scyliorhinus canicula* L.	Inhibition	No	-	Peptide	Prostate cancer (MDA-PCa 2b cells)	[123]
pE-K092D	Dogfish *Scyliorhinus canicula* L. (pyroglutamate modification of K092D peptide)						[124]
acyclolaxaphycin B	Cyanobacteria *Anabaena torulosa*	Activation	No	Mitochondria; ROS; mTOR; AMPK; p70S6	Peptide (acyclic B-type laxaphycins)	Neuroblastoma (SH-SY5Y cells)	[126]
acyclolaxaphycin B3							
[des-(Ala^4^-Hle^5^)]acyclolaxaphycin B	Gastropod *Stylocheilus striatus*.						
[des-(Ala^4^-Hle^5^)]acyclolaxaphycin B3							
PYP15	Marine alga *Pyropia yezoensis*	Inhibition	No	IGF-IR; Akt/mTOR	peptide	Mouse skeletal muscle cells (C2C12 cells)	[128]
**Lipids**							
Eicosapentaenoic acid-enriched phosphatidylcholine (EPA-PC)	Fish oil, antarctic krill, sea cucumbers	Activation	No	-	Lipid	Aβ1-42-induced neurotoxicity in vivo (rats)	[131]
Melonoside A	Marine sponge *Melonanchora kobjakovae*	Activation	No	-	ω-Glycosylated fatty acid amide	Germ cell tumor (GCT) (NCCIT-R cells)	[136]
2-*trans*-4-*trans*-decadienal	Different diatoms	Activation	No	-	Polyunsaturated aldehydes	Sea urchin embrios *Paracentrotus lividus;* lung cancer (A549 cells)	[139]
2-*trans*-4-*trans*-7-octadienal							
2-*trans*-4-*trans*-7-heptadienal							
**Lectins**							
*Ulva pertusa* lectin 1 (the expression cassette the lectin integrated in the adenovirus genome)	Marine alga *Ulva pertusa*	Activation	No	-	Lectin	Liver cancer (BEL-7404 and Huh7 cells)	[140]
Halilectin-3	Marine sponge *Haliclona caerulea*	Activation	No	-	Lectin	Breast cancer (MCF7 cells)	[142]
**Polysaccharides**							
3,6-*O*-sulfated chitosan	Marine shrimps	Inhibition	No	PI3K/Akt/mTOR pathway	Sulfated polysaccharide	Cervical carcinoma (HeLa cells)	[143]
Fucoidan	*Fucus vesiculosus* and other brown algae	Inhibition of cytotoxic CCl4-induced autophagy	No	TGF-β1/Smad pathway	Sulfated polysaccharide	In vivo CCl_4_- and BDL-induced liver fibrosis	[146]
**Other metabolites**							
K41 A	Marine actinobacterium *Streptomyces cacao*	Inhibition (contradictive results are reported)	No (contradictive results are reported)	-	Polyether antibiotic	Cervical cancer (HeLa cells); prostate cancer (PC-3 cells); colorectal cancer (CaCo-2 cells)	[147]
29-*O*-methyl-K41 A				-			
Zosteropenillines A–L	Marine-derived fungus *Penicillium thomii*	Inhibition	No	-	Polyketide	Prostate cancer (PC-3 cells)	[148]
Diphlorethohydroxycarmalol (DPHC)	Marine alga *Ishige okamurae*	Inhibition of the particulate matter-induced autophagy	No	-	Polyphenol	Non-cancer keratinocytes (HaCaT cells)	[150]

^1^ Effect validated using several independent methods (according to the Guidelines by Klionsky at al. [7,8,30,31]); ^2^ Direct or indirect target of the compound suggested to be related to the effect on autophagy.

## 4. Autophagy-Modulatory Effect of the Compounds with an Undefined Structure, or of the Compounds Mixtures

This section reports an autophagy-modulatory effect of the marine-derived compounds with an undefined structure or of the compound mixtures. Specifically, the activity of extracts of the marine organisms, or of their fractions, is discussed. Note, none of the specific effects of the extracts and fractions on autophagy described below can be considered as validated (according to the Guidelines by Klionsky et al. [7,8,30,31]). Therefore, further investigation of the active molecules as well as their autophagy-modulating activity is required. The data are summarized in Table 3.

Choi et al. have suggested that the **methanol/dichloromethane extract of marine sponge *Lipastrotethya* sp.** contains compounds capable of autophagy induction in p53-deficient human colon cancer HCT116 p53 KO cells [151]. However, this suggestion was made exclusively based on the observed upregulation of LC3-II protein in the treated cells and, therefore, cannot be assumed as relevant evidence [7,8,30,31]. The group of Park has reported an extract of the **marine sponge *Agelas* sp.** to induce autophagy in human hepatocellular carcinoma cells [152]. This conclusion was made based solely on the altered expression of several autophagy-related proteins in the cells treated with the sponge extract. Hence, further validating experiments are required. Yu et al. have described a strain of **marine bacteria *Streptomyces* sp.** U3 isolated from mangrove [153]. The crude extract of this bacteria possesses an algicidal activity and could inhibit a growth of a harmful marine alga *Heterosigma akashiwo*. However, the active compounds were not isolated and identified. Based on electron microscopy data, the authors suggested that the compounds presented in the crude extract of *Streptomyces* sp. U3 could induce autophagy of the algal cells [153]. As no further validation experiments were performed, this speculation awaits further verification.

Castro-Carvalho et al. have reported the extracts of two **marine-derived fungi**, namely, ***Neosartorya tsunodae* KUFC 9213** and ***Neosartorya laciniosa* KUFC 7896** to induce autophagy in non-small-cell lung cancer cells A459 when combined with doxorubicin [154]. This conclusion was made exclusively based on the observation of an increased number of autophagosome-like vesicles in the treated cells and requires extensive validation and verification. No individual compounds responsible for the suggested activity of the extracts were identified. Leri et al. have described the ability of extract of the **seagrass Posidonia oceanica (L.) Delile** to modulate (induce) autophagy in human fibrosarcoma HT1080 cells [155]. This conclusion was made based on the increased autophagosome number in the treated cells. Hence, further careful examination of the effect on autophagy along with an active compound identification is required. The group of Nam investigated the effect of **crude protein extract of an edible alga *Pyropia yezoensis*** on the dexamethasone-induced skeletal muscle atrophy (myotube atrophy) using the model of mouse skeletal muscle C2C12 cells [129]. It was reported that the *P. yezoensis* crude protein extract can inhibit dexamethasone-induced conversion (lipidation) of LC3B-I into LC3B-II. No further validation experiments have been performed. Of note, later, the same group examined the activity of the individual peptide PYP15 isolated from this alga [127] (see Section 3.4.). Galasso and colleagues reported the isolation of a **glycoprotein-containing fraction from the phenol-water extract of marine dinoflagellate *Alexandrium minutum*** [156]. The isolated fraction was capable of selective killing of human lung adenocarcinoma A549 cells and induced the upregulation of several autophagy- and mitophagy-related genes. Thus, the authors suggested the activation of mitophagy in the treated cells [156]. However, further analysis of the relevant protein expression is required to validate the above-described speculation.

**Table 3:** Autophagy-modulatory effect of the marine-derived compounds with undefined structure, or of the compounds mixtures ^1^.

Name	Source organism	Suggested effect on autophagy	Effect validated? ^1^	Target ^2^	Molecular Class	Model	Ref.
Glycoprotein-containing fraction from *Alexandrium minutum*	Marine dinoflagellate *Alexandrium minutum*	Activation of mitophagy	No	-	Glycoprotein (?)	Lung adenocarcinoma (A549 cells)	[156]
*Pyropia yezoensis* crude protein extract	Marine alga *Pyropia yezoensis*	Inhibition	No	-	-	Mouse skeletal muscle cells (C2C12 cells)	[129]
Extract of *Posidonia oceanica* (L.) Delile	Seagrass *Posidonia oceanica* (L.) Delile	Activation	No	-	-	Fibrosarcoma (HT1080 cells)	[155]
Extract of *Neosartorya tsunodae* KUFC 9213	Marine fungi *Neosartorya tsunodae* KUFC 9213	Activation	No	-	-	Nonsmall cell lung cancer (A459 cells)	[154]
Extract of *Neosartorya laciniosa* KUFC 7896	Marine fungi *Neosartorya laciniosa* KUFC 7896						
Extract of *Streptomyces* sp. U3	Marine bacteria *Streptomyces* sp. U3/mangrove	Activation	No	-	-	Marine alga *Heterosigma akashiwo*	[153]
Extract of *Agelas* sp.	Marine sponge *Agelas* sp.	Activation	No	ER stress, ROS; IRE1α; CHOP; ATF4; JNK	-	Hepatocellular carcinoma (Hep3B cells)	[152]
Extract of *Lipastrotethya* sp.	Marine sponge *Lipastrotethya* sp.	Activation	No	-	-	Colon cancer (HCT116 p53 KO cells)	[151]

^1^ Effect validated using several independent methods (according to the Guidelines by Klionsky at al. [7,8,30,31]); ^2^ Direct or indirect target of the compound suggested to be related to the effect on autophagy.

## 5. Concluding Remarks

The current review covers the scientific literature related to the Blue-print autophagy topic published between the beginning of 2016 and August 2020. Over this time, 62 marine-derived compounds have been reported to possess an autophagy-modulatory activity. It can be postulated that all these molecules indeed affect autophagy, as for the vast majority of them, an alteration of the established autophagy markers LC3-II and SQSTM1 was observed. However, for 59% of the substances (38/62), the suggested activity, i.e., activation or inhibition, has not been properly validated and, therefore, unfortunately, cannot be assumed. This is a highly important observation, which shall be considered as a warning and should promote a careful “double-check” of the already published data before they can be used as a basement for further hypotheses. It is very likely that a similar situation might appear in the adjacent fields of science, which not only deal with naturally derived substances but also with synthetic autophagy modulators.

Cancer cell lines are the model that was almost exclusively used in the studies reviewed in the current manuscript. Therefore, it is rather likely that a significant proportion of the molecules bearing cytotoxic properties and described above induces cytoprotective autophagy as a “side effect” of its cytotoxic action on cancer cells. Nevertheless, this speculation requires extensive validation in every single case. On the other hand, the ability to inhibit autophagy or to induce cytotoxic autophagy is also rather frequently reported—i.e., 25% (6/24) and 21% (5/24), correspondently, referring to the group of autophagy modulators with validated effects (Table 1).

Marine sponges were identified as the main source of the active compounds, reviewed in the current manuscript (29%, 18/62; Table 1 and Table 2). These animals are known to produce a variety of bioactive molecules often bearing unique chemical structure and possessing pronounced biological activities [14,157]. 16% (10/62) of the compounds were obtained from marine bacteria and 6.5% (4/62) from marine-derived fungi. Surprisingly, a significant proportion of the compounds, namely 14% (9/62), was obtained from alga. Chemically, the compounds could be assigned to the various chemical classes; most of them belong to alkaloids, macrocyclic molecules, triterpenes, and peptides.

Finally, it should be noted that, in recent years, it has become clear that autophagy does interact with basically every biochemical process in the living cells [9]. Therefore, one should expect that the drugs affecting any other biological targets, and especially cytotoxic molecules, will also directly or non-directly affect autophagy. Hence, it is highly likely that the “autophagy-modulating activity” can actually be assigned to the most (if not all) of biologically active natural and synthetic compounds. In other words, the only reason why the autophagy-modulatory activity has not been reported so far for the majority of bioactive molecules is that it has not been examined yet.

## References

[1] Mizushima N., Komatsu M. (2011). Autophagy: Renovation of cells and tissues. Cell.

[2] Yang Z.J., Chee C.E., Huang S., Sinicrope F.A. (2011). The role of autophagy in cancer: Therapeutic implications. Mol. Cancer Ther..

[3] Mathew R., Karantza-Wadsworth V., White E. (2007). Role of autophagy in cancer. Nat. Rev. Cancer.

[4] Cheng Y., Ren X., Hait W.N., Yang J.M. (2013). Therapeutic targeting of autophagy in disease: Biology and pharmacology. Pharmacol. Rev..

[5] Rubinsztein D.C., Frake R.A. (2016). Yoshinori Ohsumi’s Nobel Prize for mechanisms of autophagy: From basic yeast biology to therapeutic potential. J. R. Coll. Physicians Edinb..

[6] Gewirtz D.A. (2014). The four faces of autophagy: Implications for cancer therapy. Cancer Res..

[7] Klionsky D.J., Abdelmohsen K., Abe A., Abedin M.J., Abeliovich H., Acevedo Arozena A., Adachi H., Adams C.M., Adams P.D., Adeli K. (2016). Guidelines for the use and interpretation of assays for monitoring autophagy (3rd edition). Autophagy.

[8] Klionsky D.J., Abdel-Aziz A.K., Abel S., Adamopoulos I.E., Adolph T., Agnello M., Agostinis P., Aits S., Aizawa S., Al-Abd A.M. (2020). Guidelines for the use and interpretation of assays for monitoring autophagy (4rd edition). Autophagy.

[9] Klionsky D.J. (2020). Autophagy participates in, well, just about everything. Cell Death Differ..

[10] Solitro A.R., MacKeigan J.P. (2016). Leaving the lysosome behind: Novel developments in autophagy inhibition. Future Med. Chem..

[11] Bergmann W., Burke D.C. (1956). Contributions to the Study of Marine Products. XL. The Nucleosides of Sponges.1 IV. Spongosine2. J. Org. Chem..

[12] Bergmann W., Feeney R.J. (1951). Contributions to the study of marine products. XXXII. The nucleosides of spongies. I. J. Org. Chem..

[13] Bergmann W., Stempien M.F. (1957). Contributions to the Study of Marine Products. XLIII. The Nucleosides of Sponges. V. The Synthesis of Spongosine1. J. Org. Chem..

[14] Stonik V. (2009). Marine natural products: A way to new drugs. Acta Nat..

[15] Molinski T.F., Dalisay D.S., Lievens S.L., Saludes J.P. (2009). Drug development from marine natural products. Nat. Rev. Drug Discov.

[16] Newman D.J., Cragg G.M. (2004). Marine natural products and related compounds in clinical and advanced preclinical trials. J. Nat. Prod..

[17] Newman D.J., Cragg G.M. (2014). Marine-sourced anti-cancer and cancer pain control agents in clinical and late preclinical development. Mar. Drugs.

[18] Galmarini C.M., D’Incalci M., Allavena P. (2014). Trabectedin and plitidepsin: Drugs from the sea that strike the tumor microenvironment. Mar. Drugs.

[19] Dyshlovoy S.A., Honecker F. (2018). Marine Compounds and Cancer: 2017 Updates. Mar. Drugs.

[20] Dyshlovoy S.A., Honecker F. (2019). Marine Compounds and Cancer: The First Two Decades of XXI Century. Mar. Drugs.

[21] Dyshlovoy S.A., Honecker F. (2015). Marine Compounds and Cancer: Where Do We Stand?. Mar. Drugs.

[22] Ruocco N., Costantini S., Costantini M. (2016). Blue-Print Autophagy: Potential for Cancer Treatment. Mar. Drugs.

[23] Dyshlovoy A.S., Honecker F. (2018). Marine Compounds and Autophagy: Beginning of a New Era. Mar. Drugs.

[24] Dyshlovoy S.A., Honecker F. (2020). Marine Drugs Acting as Autophagy Modulators. Mar. Drugs.

[25] Carr G., Williams D.E., Díaz-Marrero A.R., Patrick B.O., Bottriell H., Balgi A.D., Donohue E., Roberge M., Andersen R.J. (2010). Bafilomycins Produced in Culture by Streptomyces spp. Isolated from Marine Habitats Are Potent Inhibitors of Autophagy. J. Nat. Prod..

[26] PubMed. U.S National Library of Medicine, National Institutes of Health. Search: Autophagy. https://www.ncbi.nlm.nih.gov/pubmed/?term=autophagy.

[27] PubMed. U.S. National Library of Medicine, National Institutes of Health Search: “Autophagy” and “Marine”. https://pubmed.ncbi.nlm.nih.gov/?term=%28Autophagy%5BTitle%2FAbstract%5D%29+AND+%28marine%5BTitle%2FAbstract%5D%29&sort=date&size=50.

[28] Yoshii S.R., Mizushima N. (2017). Monitoring and Measuring Autophagy. Int. J. Mol. Sci..

[29] Tanida I., Ueno T., Kominami E. (2008). LC3 and Autophagy. Methods Mol. Biol..

[30] Klionsky D.J., Abeliovich H., Agostinis P., Agrawal D.K., Aliev G., Askew D.S., Baba M., Baehrecke E.H., Bahr B.A., Ballabio A. (2008). Guidelines for the use and interpretation of assays for monitoring autophagy in higher eukaryotes. Autophagy.

[31] Klionsky D.J., Abdalla F.C., Abeliovich H., Abraham R.T., Acevedo-Arozena A., Adeli K., Agholme L., Agnello M., Agostinis P., Aguirre-Ghiso J.A. (2012). Guidelines for the use and interpretation of assays for monitoring autophagy. Autophagy.

[32] Kung H.-J., Changou C., Nguyen H.G., Yang J.C., Evans C.P., Bold R.J., Chuang F., Tindal D.J. (2013). Autophagy and prostate cancer therapeutics. Prostate Cancer.

[33] Klionsky D.J. (2019). 2020 Is not that far away, which means it is time for the new guidelines. Autophagy.

[34] Roll D.M., Ireland C.M., Lu H.S.M., Clardy J. (1988). Fascaplysin, an unusual antimicrobial pigment from the marine sponge Fascaplysinopsis sp. J. Org. Chem..

[35] Bharate S.B., Manda S., Mupparapu N., Battini N., Vishwakarma R.A. (2012). Chemistry and biology of fascaplysin, a potent marine-derived CDK-4 inhibitor. Mini Rev. Med. Chem..

[36] Lin J., Yan X.J., Chen H.M. (2007). Fascaplysin, a selective CDK4 inhibitor, exhibit anti-angiogenic activity in vitro and in vivo. Cancer Chemother. Pharmacol..

[37] Oh T.I., Lee Y.M., Nam T.J., Ko Y.S., Mah S., Kim J., Kim Y., Reddy R.H., Kim Y.J., Hong S. (2017). Fascaplysin Exerts Anti-Cancer Effects through the Downregulation of Survivin and HIF-1α and Inhibition of VEGFR2 and TRKA. Int. J. Mol. Sci..

[38] Meng N., Mu X., Lv X., Wang L., Li N., Gong Y. (2019). Autophagy represses fascaplysin-induced apoptosis and angiogenesis inhibition via ROS and p8 in vascular endothelia cells. Biomed. Pharmacother..

[39] Florean C., Schnekenburger M., Lee J.Y., Kim K.R., Mazumder A., Song S., Kim J.M., Grandjenette C., Kim J.G., Yoon A.Y. (2016). Discovery and characterization of Isofistularin-3, a marine brominated alkaloid, as a new DNA demethylating agent inducing cell cycle arrest and sensitization to TRAIL in cancer cells. Oncotarget.

[40] Bechmann N., Ehrlich H., Eisenhofer G., Ehrlich A., Meschke S., Ziegler C.G., Bornstein S.R. (2018). Anti-Tumorigenic and Anti-Metastatic Activity of the Sponge-Derived Marine Drugs Aeroplysinin-1 and Isofistularin-3 against Pheochromocytoma In Vitro. Mar. Drugs.

[41] Teeyapant R., Kreis P., Wray V., Witte L., Proksch P. (1993). Brominated Secondary Compounds from the Marine Sponge Verongia aerophoba and the Sponge Feeding Gastropod Tylodina perversa. Z. Für Nat. C.

[42] Medina R.A., Goeger D.E., Hills P., Mooberry S.L., Huang N., Romero L.I., Ortega-Barría E., Gerwick W.H., McPhail K.L., Coibamide A. (2008). A, a potent antiproliferative cyclic depsipeptide from the Panamanian marine cyanobacterium Leptolyngbya sp. J. Am. Chem. Soc..

[43] Luesch H., Yoshida W.Y., Moore R.E., Paul V.J., Corbett T.H. (2001). Total structure determination of apratoxin A, a potent novel cytotoxin from the marine cyanobacterium Lyngbya majuscula. J. Am. Chem. Soc..

[44] Serrill J.D., Wan X., Hau A.M., Jang H.S., Coleman D.J., Indra A.K., Alani A.W., McPhail K.L., Ishmael J.E. (2016). Coibamide A, a natural lariat depsipeptide, inhibits VEGFA/VEGFR2 expression and suppresses tumor growth in glioblastoma xenografts. Investig. New Drugs.

[45] Wan X., Serrill J.D., Humphreys I.R., Tan M., McPhail K.L., Ganley I.G., Ishmael J.E. (2018). ATG5 Promotes Death Signaling in Response to the Cyclic Depsipeptides Coibamide A and Apratoxin A. Mar. Drugs.

[46] Wang C., Niederstrasser H., Douglas P.M., Lin R., Jaramillo J., Li Y., Oswald N.W., Zhou A., McMillan E.A., Mendiratta S. (2017). Small-molecule TFEB pathway agonists that ameliorate metabolic syndrome in mice and extend C. elegans lifespan. Nat. Commun..

[47] Jomon K., Kuroda Y., Ajisaka M., Sakai H. (1972). A new antibiotic, ikarugamycin. J. Antibiot..

[48] Urdiales J., Morata P., De Castro I.N., Sánchez-Jiménez F. (1996). Antiproliferative effect of dehydrodidemnin B (DDB), a depsipeptide isolated from Mediterranean tunicates. Cancer Lett..

[49] Losada A., Berlanga J.J., Molina-Guijarro J.M., Jiménez-Ruiz A., Gago F., Avilés P., de Haro C., Martínez-Leal J.F. (2020). Generation of endoplasmic reticulum stress and inhibition of autophagy by plitidepsin induces proteotoxic apoptosis in cancer cells. Biochem. Pharmacol..

[50] Wright A.E., Botelho J.C., Guzmán E., Harmody D., Linley P., McCarthy P.J., Pitts T.P., Pomponi S.A., Reed J.K. (2007). Neopeltolide, a macrolide from a lithistid sponge of the family Neopeltidae. J. Nat. Prod..

[51] Fuwa H., Sato M. (2017). A Synthetic Analogue of Neopeltolide, 8,9-Dehydroneopeltolide, Is a Potent Anti-Austerity Agent against Starved Tumor Cells. Mar. Drugs.

[52] Girard M., Bélanger J., ApSimon J.W., Garneau F.-X., Harvey C., Brisson J.-R., Frondoside A. (1990). A novel triterpene glycoside from the holothurian *Cucumaria frondosa*. Can. J. Chem..

[53] Dyshlovoy S.A., Menchinskaya E.S., Venz S., Rast S., Amann K., Hauschild J., Otte K., Kalinin V.I., Silchenko A.S., Avilov S.A. (2016). The marine triterpene glycoside frondoside A exhibits activity in vitro and in vivo in prostate cancer. Int. J. Cancer.

[54] Dyshlovoy S.A., Madanchi R., Hauschild J., Otte K., Alsdorf W.H., Schumacher U., Kalinin V.I., Silchenko A.S., Avilov S.A., Honecker F. (2017). The marine triterpene glycoside frondoside A induces p53-independent apoptosis and inhibits autophagy in urothelial carcinoma cells. BMC Cancer.

[55] Dyshlovoy S., Rast S., Hauschild J., Otte K., Alsdorf W., Madanchi R., Kalinin V., Silchenko A., Avilov S., Dierlamm J. (2017). Frondoside A induces AIF-associated caspase-independent apoptosis in Burkitt’s lymphoma cells. Leuk. Lymphoma.

[56] Wu H.-Y., Yang F.-L., Li L.-H., Rao Y.K., Ju T.-C., Wong W.-T., Hsieh C.-Y., Pivkin M.V., Hua K.-F., Wu S.-H. (2018). Ergosterol peroxide from marine fungus Phoma sp. induces ROS-dependent apoptosis and autophagy in human lung adenocarcinoma cells. Sci. Rep..

[57] Ravi B.N., Wells R.J., Croft K.D. (1981). Malabaricane triterpenes from a Fijian collection of the sponge Jaspis stellifera. J. Org. Chem..

[58] Wang R., Zhang Q., Peng X., Zhou C., Zhong Y., Chen X., Qiu Y., Jin M., Gong M., Kong D. (2016). Stellettin B Induces G1 Arrest, Apoptosis and Autophagy in Human Non-small Cell Lung Cancer A549 Cells via Blocking PI3K/Akt/mTOR Pathway. Sci. Rep..

[59] Satake M., MacKenzie L., Yasumoto T. (1997). Identification of Protoceratium reticulatum as the biogenetic origin of yessotoxin. Nat. Toxins.

[60] Draisci R., Ferretti E., Palleschi L., Marchiafava C., Poletti R., Milandri A., Ceredi A., Pompei M. (1999). High levels of yessotoxin in mussels and presence of yessotoxin and homoyessotoxin in dinoflagellates of the Adriatic Sea. Toxicon.

[61] Rubiolo J.A., López-Alonso H., Martínez P., Millán A., Cagide E., Vieytes M.R., Vega F.V., Botana L.M. (2014). Yessotoxin induces ER-stress followed by autophagic cell death in glioma cells mediated by mTOR and BNIP3. Cell. Signal..

[62] Korsnes M.S., Røed S.S., Tranulis M.A., Espenes A., Christophersen B. (2014). Yessotoxin triggers ribotoxic stress. Toxicol. Vitr..

[63] Korsnes M.S., Kolstad H., Kleiveland C.R., Korsnes R., Ørmen E. (2016). Autophagic activity in BC3H1 cells exposed to yessotoxin. Toxicol Vitr..

[64] Molinski T.F., Makarieva T.N., Stonik V.A. (2000). (-)-Rhizochalin is a dimeric enantiomorphic (2R)-sphingolipid: Absolute configuration of pseudo-C(2v)-symmetric bis-2-amino-3-alkanols by CD. Angew. Chem. Int. Ed. Engl..

[65] Makarieva T.N., Denisenko V.A., Stonik V.A., Milgrom Y.M., Rashkes Y.V. (1989). Rhizochalin, a novel secondary metabolite of mixed biosynthesis from the sponge *Rhizochalina Incrustata*. Tetrahedron Lett..

[66] Makarieva T.N., Zakharenko A.M., Denisenko V.A., Dmitrenok P.S., Guzii A.G., Shubina L.K., Kapustina I.I., Fedorov S.N. (2007). Rhizochalinin A, a new antileukemic two-headed sphingolipid from the sponge *Rhizochalina incrustata*. Chem. Nat. Compd..

[67] Fedorov S.N., Makarieva T.N., Guzii A.G., Shubina L.K., Kwak J.Y., Stonik V.A. (2009). Marine two-headed sphingolipid-like compound rhizochalin inhibits EGF-induced transformation of JB6 P^+^ Cl41 cells. Lipids.

[68] Khanal P., Kang B.S., Yun H.J., Cho H.G., Makarieva T.N., Choi H.S. (2011). Aglycon of rhizochalin from the *Rhizochalina incrustata* induces apoptosis via activation of AMP-activated protein kinase in HT-29 colon cancer cells. Biol. Pharm. Bull..

[69] Dyshlovoy S.A., Otte K., Tabakmakher K.M., Hauschild J., Makarieva T.N., Shubina L.K., Fedorov S.N., Bokemeyer C., Stonik V.A., von Amsberg G. (2018). Synthesis and anticancer activity of the derivatives of marine compound rhizochalin in castration resistant prostate cancer. Oncotarget.

[70] Dyshlovoy S.A., Otte K., Alsdorf W.H., Hauschild J., Lange T., Venz S., Bauer C.K., Bahring R., Amann K., Mandanchi R. (2016). Marine compound rhizochalinin shows high in vitro and in vivo efficacy in castration resistant prostate cancer. Oncotarget.

[71] Searle P.A., Molinski T.F. (1995). Trachycladines A and B: 2’-C-methyl-5’-deoxyribofuranosyl nucleosides from the marine sponge Trachycladus laevispirulifer. J. Org. Chem..

[72] Ichiba T., Nakao Y., Scheuer P.J., Sata N.U., Kelly-Borges M. (1995). Kumusine, a chloroadenine riboside from a sponge, Theonella sp. Tetrahedron Lett..

[73] Peitsinis Z.V., Mitrakas A.G., Nakiou E.A., Melidou D.A., Kalamida D., Kakouratos C., Koukourakis M.I., Koumbis A.E. (2017). Trachycladines and Analogues: Synthesis and Evaluation of Anticancer Activity. ChemMedChem.

[74] Wakabayashi T., Kageyama-Kawase R., Naruse N., Funahashi Y., Yoshimatsu K. (2000). Luminacins: A family of capillary tube formation inhibitors from Streptomyces sp. II. Biological activities. J. Antibiot..

[75] Shin Y.S., Cha H.Y., Lee B.-S., Kang S.U., Hwang H.S., Kwon H.C., Kim C.-H., Choi E.C. (2016). Anti-cancer Effect of Luminacin, a Marine Microbial Extract, in Head and Neck Squamous Cell Carcinoma Progression via Autophagic Cell Death. Cancer Res. Treat. Off. J. Korean Cancer Assoc..

[76] Peng J., Yuan J.P., Wu C.F., Wang J.H. (2011). Fucoxanthin, a marine carotenoid present in brown seaweeds and diatoms: Metabolism and bioactivities relevant to human health. Mar. Drugs.

[77] Long Y., Cao X., Zhao R., Gong S., Jin L., Feng C. (2020). Fucoxanthin treatment inhibits nasopharyngeal carcinoma cell proliferation through induction of autophagy mechanism. Environ. Toxicol..

[78] Zhang L., Wang H., Fan Y., Gao Y., Li X., Hu Z., Ding K., Wang Y., Wang X. (2017). Fucoxanthin provides neuroprotection in models of traumatic brain injury via the Nrf2-ARE and Nrf2-autophagy pathways. Sci. Rep..

[79] Liao G., Gao B., Gao Y., Yang X., Cheng X., Ou Y. (2016). Phycocyanin Inhibits Tumorigenic Potential of Pancreatic Cancer Cells: Role of Apoptosis and Autophagy. Sci. Rep..

[80] Yu H., Wu C.-L., Wang X., Ban Q., Quan C., Liu M., Dong H., Li J., Kim G.-Y., Choi Y.H. (2019). SP600125 enhances C-2-induced cell death by the switch from autophagy to apoptosis in bladder cancer cells. J. Exp. Clin. Cancer Res..

[81] Cingolani F., Simbari F., Abad J.L., Casasampere M., Fabrias G., Futerman A.H., Casas J. (2017). Jaspine B induces nonapoptotic cell death in gastric cancer cells independently of its inhibition of ceramide synthase. J. Lipid Res..

[82] Kuroda I., Musman M., Ohtani I.I., Ichiba T., Tanaka J., Gravalos D.G., Higa T. (2002). Pachastrissamine, a Cytotoxic Anhydrophytosphingosine from a Marine Sponge, Pachastrissa sp. J. Nat. Prod..

[83] Zhang E., Wang S., Li L.-L., Hua Y.-G., Yue J.-F., Li J.-F., Jin C.-Y. (2018). Discovery of novel jaspine B analogues as autophagy inducer. Biorg. Med. Chem. Lett..

[84] Miyamoto M., Kawamatsu Y., Kawashima K., Shinohara M., Tanaka K., Tatsuoka S., Nakanishi K. (1967). Chromomycin A2, A3 and A4. Tetrahedron.

[85] Quiñoà E., Crews P. (1987). Phenolic constituents of Psammaplysilla. Tetrahedron Lett..

[86] Luibrand R.T., Erdman T.R., Vollmer J.J., Scheuer P.J., Finer J., Clardy J. (1979). Ilimaquinone, a sesquiterpenoid quinone from a marine sponge. Tetrahedron.

[87] Ratovitski E.A. (2016). Tumor Protein (TP)-p53 Members as Regulators of Autophagy in Tumor Cells upon Marine Drug Exposure. Mar. Drugs.

[88] Ha Y.N., Song S., Orlikova-Boyer B., Cerella C., Christov C., Kijjoa A., Diederich M. (2020). Petromurin C Induces Protective Autophagy and Apoptosis in FLT3-ITD-Positive AML: Synergy with Gilteritinib. Mar. Drugs.

[89] Buttachon S., Ramos A.A., Inácio Â., Dethoup T., Gales L., Lee M., Costa P.M., Silva A.M.S., Sekeroglu N., Rocha E. (2018). Bis-Indolyl Benzenoids, Hydroxypyrrolidine Derivatives and Other Constituents from Cultures of the Marine Sponge-Associated Fungus Aspergillus candidus KUFA0062. Mar. Drugs.

[90] Ooike M., Nozawa K., Kawai K.-I., Udagawa S.-i. (1997). Bisindolylbenzenoids from ascostromata of Petromycesmuricatus. Can. J. Chem..

[91] Mauvezin C., Neufeld T.P. (2015). Bafilomycin A1 disrupts autophagic flux by inhibiting both V-ATPase-dependent acidification and Ca-P60A/SERCA-dependent autophagosome-lysosome fusion. Autophagy.

[92] Sharma S., Guru S.K., Manda S., Kumar A., Mintoo M.J., Prasad V.D., Sharma P.R., Mondhe D.M., Bharate S.B., Bhushan S. (2017). A marine sponge alkaloid derivative 4-chloro fascaplysin inhibits tumor growth and VEGF mediated angiogenesis by disrupting PI3K/Akt/mTOR signaling cascade. Chem. Biol. Interact..

[93] Hubbard R., Rimington C. (1950). The biosynthesis of prodigiosin, the tripyrrylmethene pigment from Bacillus prodigiosus (Serratia marcescens). Biochem. J..

[94] Williamson N.R., Fineran P.C., Leeper F.J., Salmond G.P. (2006). The biosynthesis and regulation of bacterial prodiginines. Nat. Rev. Microbiol.

[95] Bennett J.W., Bentley R. (2000). Seeing Red: The Story of Prodigiosin.

[96] Cheng S.Y., Chen N.F., Kuo H.M., Yang S.N., Sung C.S., Sung P.J., Wen Z.H., Chen W.F. (2018). Prodigiosin stimulates endoplasmic reticulum stress and induces autophagic cell death in glioblastoma cells. Apoptosis.

[97] Cheng M.F., Lin C.S., Chen Y.H., Sung P.J., Lin S.R., Tong Y.W., Weng C.F. (2017). Inhibitory Growth of Oral Squamous Cell Carcinoma Cancer via Bacterial Prodigiosin. Mar. Drugs.

[98] Castellano I., Seebeck F.P. (2018). On ovothiol biosynthesis and biological roles: From life in the ocean to therapeutic potential. Nat. Prod. Rep..

[99] Brancaccio M., Russo M., Masullo M., Palumbo A., Russo G.L., Castellano I. (2019). Sulfur-containing histidine compounds inhibit γ-glutamyl transpeptidase activity in human cancer cells. J. Biol. Chem..

[100] Sousa M.L., Preto M., Vasconcelos V., Linder S., Urbatzka R. (2019). Antiproliferative Effects of the Natural Oxadiazine Nocuolin A Are Associated With Impairment of Mitochondrial Oxidative Phosphorylation. Front. Oncol..

[101] Kita Y., Fujioka H. (2012). Marine pyrroloiminoquinone alkaloids. Top. Curr. Chem..

[102] Cowan J., Shadab M., Nadkarni D.H., Kc K., Velu S.E., Yusuf N. (2019). A Novel Marine Natural Product Derived Pyrroloiminoquinone with Potent Activity against Skin Cancer Cells. Mar. Drugs.

[103] Wu C.-F., Lee M.-G., El-Shazly M., Lai K.-H., Ke S.-C., Su C.-W., Shih S.-P., Sung P.-J., Hong M.-C., Wen Z.-H. (2018). Isoaaptamine Induces T-47D Cells Apoptosis and Autophagy via Oxidative Stress. Mar. Drugs.

[104] Tsukamoto S., Yamanokuchi R., Yoshitomi M., Sato K., Ikeda T., Rotinsulu H., Mangindaan R.E.P., de Voogd N.J., van Soest R.W.M., Yokosawa H. (2010). Aaptamine, an alkaloid from the sponge *Aaptos suberitoides*, functions as a proteasome inhibitor. Biorg. Med. Chem. Lett..

[105] Shubina L.K., Kalinovsky A.I., Fedorov S.N., Radchenko O.S., Denisenko V.A., Dmitrenok P.S., Dyshlovoy S.A., Krasokhin V.B., Stonik V.A. (2009). Aaptamine alkaloids from the vietnamese sponge *Aaptos* sp. Nat. Prod. Commun..

[106] Shubina L.K., Makarieva T.N., Dyshlovoy S.A., Fedorov S.N., Dmitrenok P.S., Stonik V.A. (2010). Three new aaptamines from the marine sponge *Aaptos* sp. and their proapoptotic properties. Nat. Prod. Commun..

[107] Weindling R. (1932). Trichoderma lignorum as a parasite of other soil fungi. Phytopathology.

[108] Park G.-B., Jeong J.-Y., Kim D. (2019). Gliotoxin Enhances Autophagic Cell Death via the DAPK1-TAp63 Signaling Pathway in Paclitaxel-Resistant Ovarian Cancer Cells. Mar. Drugs.

[109] Cimino G., De Stefano S., Minale L., Trivellone E. (1977). 12-epi-Scalarin and 12-epi-deoxoscalarin, sesterterpenes from the sponge Spongia nitens. J. Chem. Soc., Perkin Trans..

[110] Guzmán E.A., Pitts T.P., Diaz M.C., Wright A.E. (2019). The marine natural product Scalarin inhibits the receptor for advanced glycation end products (RAGE) and autophagy in the PANC-1 and MIA PaCa-2 pancreatic cancer cell lines. Investig. New Drugs.

[111] Tsai T.C., Chen H.Y., Sheu J.H., Chiang M.Y., Wen Z.H., Dai C.F., Su J.H. (2015). Structural Elucidation and Structure-Anti-inflammatory Activity Relationships of Cembranoids from Cultured Soft Corals Sinularia sandensis and Sinularia flexibilis. J. Agric. Food Chem..

[112] Tsai T.-C., Lai K.-H., Su J.-H., Wu Y.-J., Sheu J.-H. (2018). 7-Acetylsinumaximol B Induces Apoptosis and Autophagy in Human Gastric Carcinoma Cells through Mitochondria Dysfunction and Activation of the PERK/eIF2α/ATF4/CHOP Signaling Pathway. Mar. Drugs.

[113] Kobayashi M., Okamoto T., Hayashi K., Yokoyama N., Sasaki T., Kitagawa I. (1994). Marine natural products. XXXII. Absolute configurations of C-4 of the manoalide family, biologically active sesterterpenes from the marine sponge Hyrtios erecta. Chem. Pharm. Bull..

[114] Lee M.-G., Liu Y.-C., Lee Y.-L., El-Shazly M., Lai K.-H., Shih S.-P., Ke S.-C., Hong M.-C., Du Y.-C., Yang J.-C. (2018). Heteronemin, a Marine Sesterterpenoid-Type Metabolite, Induces Apoptosis in Prostate LNcap Cells via Oxidative and ER Stress Combined with the Inhibition of Topoisomerase II and Hsp90. Mar. Drugs.

[115] Weng J.-R., Chiu C.-F., Hu J.-L., Feng C.-H., Huang C.-Y., Bai L.-Y., Sheu J.-H. (2018). A Sterol from Soft Coral Induces Apoptosis and Autophagy in MCF-7 Breast Cancer Cells. Mar. Drugs.

[116] Tsai C.-R., Huang C.-Y., Chen B.-W., Tsai Y.-Y., Shih S.-P., Hwang T.-L., Dai C.-F., Wang S.-Y., Sheu J.-H. (2015). New bioactive steroids from the soft coral Klyxum flaccidum. RSC Adv..

[117] Liu M., Hansen P.E., Lin X. (2011). Bromophenols in marine algae and their bioactivities. Mar. Drugs.

[118] Guo C., Wang L., Li X., Wang S., Yu X., Xu K., Zhao Y., Luo J., Li X., Jiang B. (2019). Discovery of Novel Bromophenol–Thiosemicarbazone Hybrids as Potent Selective Inhibitors of Poly(ADP-ribose) Polymerase-1 (PARP-1) for Use in Cancer. J. Med. Chem..

[119] Wang L., Guo C., Li X., Yu X., Xu K., Jiang B., Jia X., Li C., Shi D. (2019). Design, synthesis and biological evaluation of bromophenol-thiazolylhydrazone hybrids inhibiting the interaction of translation initiation factors eIF4E/eIF4G as multifunctional agents for cancer treatment. Eur. J. Med. Chem..

[120] Wang L.J., Guo C.L., Li X.Q., Wang S.Y., Jiang B., Zhao Y., Luo J., Xu K., Liu H., Guo S.J. (2017). Discovery of Novel Bromophenol Hybrids as Potential Anticancer Agents through the Ros-Mediated Apoptotic Pathway: Design, Synthesis and Biological Evaluation. Mar. Drugs.

[121] Guo C., Wang L., Zhao Y., Jiang B., Luo J., Shi D. (2019). BOS-93, a novel bromophenol derivative, induces apoptosis and autophagy in human A549 lung cancer cells via PI3K/Akt/mTOR and MAPK signaling pathway. Exp. Ther. Med..

[122] Mokhlesi A., Stuhldreier F., Wex K.W., Berscheid A., Hartmann R., Rehberg N., Sureechatchaiyan P., Chaidir C., Kassack M.U., Kalscheuer R. (2017). Cyclic Cystine-Bridged Peptides from the Marine Sponge Clathria basilana Induce Apoptosis in Tumor Cells and Depolarize the Bacterial Cytoplasmic Membrane. J. Nat. Prod..

[123] Bosseboeuf A., Baron A., Duval E., Gautier A., Sourdaine P., Auvray P. (2019). K092A and K092B, Two Peptides Isolated from the Dogfish (Scyliorhinus canicula L.), with Potential Antineoplastic Activity Against Human Prostate and Breast Cancer Cells. Mar. Drugs.

[124] Bosseboeuf A., Baron A., Duval E., Gautier A., Sourdaine P., Auvray P. (2019). A Potential Antineoplastic Peptide of Human Prostate Cancer Cells Derived from the Lesser Spotted Dogfish (Scyliorhinus canicula L.). Mar. Drugs.

[125] Bonnard I., Rolland M., Salmon J.-M., Debiton E., Barthomeuf C., Banaigs B. (2007). Total Structure and Inhibition of Tumor Cell Proliferation of Laxaphycins. J. Med. Chem..

[126] Alvariño R., Alonso E., Bornancin L., Bonnard I., Inguimbert N., Banaigs B., Botana L.M. (2020). Biological Activities of Cyclic and Acyclic B-Type Laxaphycins in SH-SY5Y Human Neuroblastoma Cells. Mar. Drugs.

[127] Choi Y.H., Yamaguchi K., Oda T., Nam T.J. (2015). Chemical and mass spectrometry characterization of the red alga Pyropia yezoensis chemoprotective protein (PYP): Protective activity of the N-terminal fragment of PYP1 against acetaminophen-induced cell death in Chang liver cells. Int. J. Mol. Med..

[128] Lee M.-K., Choi J.-W., Choi Y.H., Nam T.-J. (2019). Protective Effect of Pyropia yezoensis Peptide on Dexamethasone-Induced Myotube Atrophy in C2C12 Myotubes. Mar. Drugs.

[129] Lee M.-K., Choi J.-W., Choi Y.H., Nam T.-J. (2018). Pyropia yezoensis Protein Prevents Dexamethasone-Induced Myotube Atrophy in C2C12 Myotubes. Mar. Drugs.

[130] Burri L., Hoem N., Banni S., Berge K. (2012). Marine omega-3 phospholipids: Metabolism and biological activities. Int. J. Mol. Sci..

[131] Wen M., Ding L., Zhang L., Zhang T., Teruyoshi Y., Wang Y., Xue C. (2019). Eicosapentaenoic Acid-Enriched Phosphatidylcholine Mitigated Aβ1-42-Induced Neurotoxicity via Autophagy-Inflammasome Pathway. J. Agric. Food Chem..

[132] Bonofiglio D., Lanzino M., Giordano C., Catalano S., Andò S., Hayat M.A. (2016). Chapter 16-Omega-3 DHA and EPA Conjugates Trigger Autophagy Through PPARγ Activation in Human Breast Cancer Cells. Autophagy: Cancer, Other Pathologies, Inflammation, Immunity, Infection, and Aging.

[133] Fukui M., Kang K.S., Okada K., Zhu B.T. (2013). EPA, an omega-3 fatty acid, induces apoptosis in human pancreatic cancer cells: Role of ROS accumulation, caspase-8 activation, and autophagy induction. J. Cell. Biochem..

[134] Gao B., Han Y.H., Wang L., Lin Y.J., Sun Z., Lu W.G., Hu Y.Q., Li J.Q., Lin X.S., Liu B.H. (2016). Eicosapentaenoic acid attenuates dexamethasome-induced apoptosis by inducing adaptive autophagy via GPR120 in murine bone marrow-derived mesenchymal stem cells. Cell Death Dis..

[135] Hsu H.C., Li S.J., Chen C.Y., Chen M.F. (2018). Eicosapentaenoic acid protects cardiomyoblasts from lipotoxicity in an autophagy-dependent manner. Cell Biol. Toxicol..

[136] Guzii A.G., Makarieva T.N., Denisenko V.A., Dmitrenok P.S., Kuzmich A.S., Dyshlovoy S.A., von Amsberg G., Krasokhin V.B., Stonik V.A. (2016). Melonoside A: An ω-Glycosylated Fatty Acid Amide from the Far Eastern Marine Sponge Melonanchora kobjakovae. Org. Lett..

[137] Caldwell G.S. (2009). The influence of bioactive oxylipins from marine diatoms on invertebrate reproduction and development. Mar. Drugs.

[138] Leflaive J., Ten-Hage L. (2009). Chemical interactions in diatoms: Role of polyunsaturated aldehydes and precursors. New Phytol..

[139] Galasso C., Celentano S., Costantini M., D’Aniello S., Ianora A., Sansone C., Romano G. (2020). Diatom-Derived Polyunsaturated Aldehydes Activate Similar Cell Death Genes in Two Different Systems: Sea Urchin Embryos and Human Cells. Int. J. Mol. Sci..

[140] Li G., Zhao Z., Wu B., Su Q., Wu L., Yang X., Chen J. (2017). Ulva pertusa lectin 1 delivery through adenovirus vector affects multiple signaling pathways in cancer cells. Glycoconj. J..

[141] Carneiro R.F., de Melo A.A., de Almeida A.S., Moura R.d.M., Chaves R.P., de Sousa B.L., do Nascimento K.S., Sampaio S.S., Lima J.P.M.S., Cavada B.S. (2013). H-3, a new lectin from the marine sponge Haliclona caerulea: Purification and mass spectrometric characterization. Int. J. Biochem. Cell Biol..

[142] do Nascimento-Neto L.G., Cabral M.G., Carneiro R.F., Silva Z., Arruda F.V.S., Nagano C.S., Fernandes A.R., Sampaio A.H., Teixeira E.H., Videira P.A. (2018). Halilectin-3, a Lectin from the Marine Sponge Haliclona caerulea, Induces Apoptosis and Autophagy in Human Breast Cancer MCF7 Cells Through Caspase-9 Pathway and LC3-II Protein Expression. Anticancer Agents Med. Chem..

[143] Gao Y., Liu W., Wang W., Zhang X., Zhao X. (2018). The inhibitory effects and mechanisms of 3,6-O-sulfated chitosan against human papillomavirus infection. Carbohydr. Polym..

[144] Usoltseva R.V., Shevchenko N.M., Malyarenko O.S., Anastyuk S.D., Kasprik A.E., Zvyagintsev N.V., Ermakova S.P. (2019). Fucoidans from brown algae Laminaria longipes and Saccharina cichorioides: Structural characteristics, anticancer and radiosensitizing activity in vitro. Carbohydr. Polym..

[145] Lin Z., Tan X., Zhang Y., Li F., Luo P., Liu H. (2020). Molecular Targets and Related Biologic Activities of Fucoidan: A Review. Mar. Drugs.

[146] Li J., Chen K., Li S., Feng J., Liu T., Wang F., Zhang R., Xu S., Zhou Y., Zhou S. (2016). Protective effect of fucoidan from Fucus vesiculosus on liver fibrosis via the TGF-β1/Smad pathway-mediated inhibition of extracellular matrix and autophagy. Drug Des. Dev. Ther..

[147] Khan N., Yılmaz S., Aksoy S., Uzel A., Tosun Ç., Kirmizibayrak P.B., Bedir E. (2019). Polyethers isolated from the marine actinobacterium Streptomyces cacaoi inhibit autophagy and induce apoptosis in cancer cells. Chem. Biol. Interact..

[148] Afiyatullov S., Leshchenko E., Berdyshev D., Sobolevskaya M., Antonov A., Denisenko V., Popov R., Pivkin M., Udovenko A., Pislyagin E. (2017). Zosteropenillines: Polyketides from the MarineDerived Fungus Penicillium thomii. Mar. Drugs.

[149] Heo S.J., Kim J.P., Jung W.K., Lee N.H., Kang H.S., Jun E.M., Park S.H., Kang S.M., Lee Y.J., Park P.J. (2008). Identification of chemical structure and free radical scavenging activity of diphlorethohydroxycarmalol isolated from a brown alga, Ishige okamurae. J. Microbiol. Biotechnol..

[150] Zhen A.X., Piao M.J., Hyun Y.J., Kang K.A., Madushan Fernando P.D.S., Cho S.J., Ahn M.J., Hyun J.W. (2019). Diphlorethohydroxycarmalol Attenuates Fine Particulate Matter-Induced Subcellular Skin Dysfunction. Mar. Drugs.

[151] Choi K., Lim H.K., Oh S.R., Chung W.-H., Jung J. (2017). Anticancer Effects of the Marine Sponge *Lipastrotethya* sp. Extract on Wild-Type and p53 Knockout HCT116 Cells. Evid.-Based Complement. Altern. Med..

[152] Choi C., Son A., Lee H.-S., Lee Y.-J., Park H.C. (2018). Radiosensitization by Marine Sponge Agelas sp. Extracts in Hepatocellular Carcinoma Cells with Autophagy Induction. Sci. Rep..

[153] Yu X., Cai G., Wang H., Hu Z., Zheng W., Lei X., Zhu X., Chen Y., Chen Q., Din H. (2018). Fast-growing algicidal Streptomyces sp. U3 and its potential in harmful algal bloom controls. J. Hazard. Mater..

[154] Castro-Carvalho B., Ramos A.A., Prata-Sena M., Malhão F., Moreira M., Gargiulo D., Dethoup T., Buttachon S., Kijjoa A., Rocha E. (2017). Marine-derived Fungi Extracts Enhance the Cytotoxic Activity of Doxorubicin in Nonsmall Cell Lung Cancer Cells A459. Pharmacogn. Res..

[155] Leri M., Ramazzotti M., Vasarri M., Peri S., Barletta E., Pretti C., Degl’Innocenti D. (2018). Bioactive Compounds from Posidonia oceanica (L.) Delile Impair Malignant Cell Migration through Autophagy Modulation. Mar. Drugs.

[156] Galasso C., Nuzzo G., Brunet C., Ianora A., Sardo A., Fontana A., Sansone C. (2018). The Marine Dinoflagellate Alexandrium minutum Activates a Mitophagic Pathway in Human Lung Cancer Cells. Mar. Drugs.

[157] Newman D.J., Cragg G.M. (2016). Natural products as sources of new drugs from 1981 to 2014. J. Nat. Prod..

